# *In Vivo* Analysis of *Lrig* Genes Reveals Redundant and Independent Functions in the Inner Ear

**DOI:** 10.1371/journal.pgen.1003824

**Published:** 2013-09-26

**Authors:** Tony del Rio, Allison M. Nishitani, Wei-Ming Yu, Lisa V. Goodrich

**Affiliations:** Department of Neurobiology, Harvard Medical School, Boston, Massachusetts, United States of America; Stanford University School of Medicine, United States of America

## Abstract

Lrig proteins are conserved transmembrane proteins that modulate a variety of signaling pathways from worm to humans. In mammals, there are three family members – Lrig1, Lrig2, and Lrig3 – that are defined by closely related extracellular domains with a similar arrangement of leucine rich repeats and immunoglobulin domains. However, the intracellular domains show little homology. Lrig1 inhibits EGF signaling through internalization and degradation of ErbB receptors. Although Lrig3 can also bind ErbB receptors *in vitro*, it is unclear whether Lrig2 and Lrig3 exhibit similar functions to Lrig1. To gain insights into *Lrig* gene functions *in vivo*, we compared the expression and function of the *Lrigs* in the inner ear, which offers a sensitive system for detecting effects on morphogenesis and function. We find that all three family members are expressed in the inner ear throughout development, with *Lrig1* and *Lrig3* restricted to subsets of cells and *Lrig2* expressed more broadly. *Lrig1* and *Lrig3* overlap prominently in the developing vestibular apparatus and simultaneous removal of both genes disrupts inner ear morphogenesis. This suggests that these two family members act redundantly in the otic epithelium. In contrast, although *Lrig1* and *Lrig2* are frequently co-expressed, *Lrig1^−/−^;Lrig2^−/−^* double mutant ears show no enhanced structural abnormalities. At later stages, *Lrig1* expression is sustained in non-sensory tissues, whereas *Lrig2* levels are enhanced in neurons and sensory epithelia. Consistent with these distinct expression patterns, *Lrig1* and *Lrig2* mutant mice exhibit different forms of impaired auditory responsiveness. Notably, *Lrig1^−/−^;Lrig2^−/−^* double mutant mice display vestibular deficits and suffer from a more severe auditory defect that is accompanied by a cochlear innervation phenotype not present in single mutants. Thus, *Lrig* genes appear to act both redundantly and independently, with *Lrig2* emerging as the most functionally distinct family member.

## Author Summary

The mammalian genome encodes three Lrig family members - Lrig1, Lrig2, and Lrig3. Lrig proteins share a characteristic extracellular domain that can bind to a variety of signaling receptors, but the three family members show little homology in the cytoplasmic domain. *Lrig1* is a tumor suppressor gene required for normal EGF signaling. Whether *Lrig2* and *Lrig3* play similar roles is not known. To address this gap in knowledge, we compared the expression and function of *Lrigs* in the mouse inner ear, which is responsible for hearing and balance. Even subtle changes in the inner ear cause easily detected deficits in hearing and balance, making it an ideal system for analysis of gene function. We find that Lrigs can act both redundantly and independently in the inner ear, with *Lrig1* and *Lrig3* cooperating to control morphogenesis and *Lrig1* and *Lrig2* acting independently to ensure proper cochlear function. However, loss of both *Lrig1* and *Lrig2* causes a more severe auditory response deficit and additionally causes a vestibular defect, suggesting some overlapping activities. Our findings provide new insights into the *in vivo* functions for the *Lrig* genes, which play important roles in vertebrate development and disease.

## Introduction

Protein-protein interactions are critical for diverse and complex biological functions throughout the animal kingdom, including nervous system development, cell adhesion and signaling, tissue morphogenesis, the immune response and human disease [1]–[4]. This functional diversity is accomplished by superfamilies of proteins harboring combinations of common protein recognition motifs. For instance, the human genome encodes hundreds of proteins with extracellular leucine rich repeats (LRR), a 20–30 amino acid motif that forms a characteristic horseshoe structure for protein-protein interactions [5], [6]. Similarly, the large immunoglobulin (Ig) superfamily of cell adhesion molecules is defined by the presence of Ig domains, which can mediate highly specific homophilic and heterophilic binding [7], [8]. Despite their abundance, LRR and Ig motifs are rarely found in the same protein, with only several dozen mammalian genes encoding LRR-Ig proteins that fall into twelve gene families [3], [9], [10]. Most of these proteins are vertebrate-specific and show discrete expression in the developing nervous system, suggesting that expansion of the LRR-Ig family may have contributed to the increased complexity of the vertebrate nervous system. Consistent with this idea, several LRR-Ig proteins have been shown to control highly specific cell-cell interactions underlying synapse formation and other aspects of nervous system development [2]. The invertebrate-specific Kekkon proteins, on the other hand, modulate signaling by binding to and downregulating EGF receptors [11], [12].

Within the *LRR-Ig* family, only the *Lrig* subfamily contains both invertebrate and vertebrate members [3], indicating that analysis of this family may provide general insights into the evolution of LRR-Ig proteins. The leucine-rich repeat and immunoglobulin-like domain proteins (Lrigs) are single pass transmembrane proteins with extracellular domains containing fifteen LRRs, three Ig-like domains and intracellular domains of varying length [13]. The fly and worm genomes each contain a single *Lrig* gene. This family is expanded in the vertebrate genome, which encodes for three family members [14]: *Lrig1* (formerly *Lig1*), *Lrig2*, and *Lrig3*. The extracellular domains are highly conserved within the family, but the cytoplasmic domains diverge significantly, with no motifs common to flies, worms, or vertebrates. This suggests that Lrig family members may interact with similar binding partners yet ultimately exert distinct downstream effects.

Most of what is known about Lrig function has come from analysis of Lrig1, which is downregulated in several human cancers [15]. Consistent with its proposed role as a tumor suppressor gene, *Lrig1* can control the activity of several receptor tyrosine kinases (rTKs) with important effects on cell proliferation and survival. For instance, Lrig1 negatively regulates members of the ErbB family of receptors by promoting receptor degradation [16]–[18]. In support of this, Lrig1 regulates EGFR levels in primary human keratinocytes [19], and loss of *Lrig1* results in increased EGF signaling and excess intestinal stem cell proliferation, tumor formation and psoriasis-like hyperplasia in mice [20]–[22]. However, Lrig1 can also inhibit Met and Ret rTK activation [23], [24], suggesting that Lrig1 activity extends beyond regulation of EGF signaling. How any Lrig protein functions at the molecular level remains a mystery.

Whether Lrig3 shares some properties with Lrig1 remains an open question. As predicted by homology in their extracellular domains, both Lrig1 and Lrig3 can bind to ErbB receptors [25]. However, although downregulation of *Lrig3* in human glioma cells caused enhanced EGFR levels [26], more recent studies indicate that Lrig3 actually opposes Lrig1's effects on EGF signaling [18]. In addition, similar to Lrig1's ability to interact with a variety of receptors, Lrig3 also binds to FGF receptors and regulates FGF and Wnt signaling in *Xenopus* [27]. Whereas several phenotypes reported in *Lrig1* mutant mice have been associated with changes in EGF signaling, loss of *Lrig3* leads to a disruption in the three-dimensional structure of the inner ear that is not easily explained by altered ErbB signaling [25], [28]. Thus, it is not yet clear how the functions identified for Lrig1 and Lrig3 *in vitro* translate to their actions *in vivo*.

Comparison of *Lrig1* and *Lrig2*, on the other hand, has suggested key differences. First, reduction of *Lrig2* either lowers or has no effect on EGFR levels *in vitro* [18], [29]. Consistent with this observation, *Lrig2* does not behave like a typical tumor suppressor in humans. For instance, *Lrig2* expression can be increased in some human tumors, and a combination of high levels of *Lrig2* and low levels of *Lrig1* correlates with a poor prognosis for a type of early-stage squamous cell carcinoma [30]. Similarly, overexpression of *Lrig2* correlates with invasiveness of pituitary adenoma [31]. In addition, studies of Lrig protein expression in human tumors have revealed fundamental differences in the subcellular distribution of these family members [32], [33]. Although *Lrig2* phenotypes have not yet been described in mice, loss of *LRIG2* causes Urofacial Syndrome in humans, which is characterized by abnormal bladder function and altered facial expression, possibly due to abnormal innervation [34].

In order to clarify whether *Lrig* genes mediate common biological functions *in vivo*, we have taken a genetic approach in mice. We have focused our analysis on the development and function of the inner ear, an exquisitely complex structure whose perfect form and function is crucial for the senses of hearing and balance [35], [36]. The spiral-shaped cochlea mediates the sense of hearing. Head position and motion is sensed by movement of fluid within the vestibular system, which consists of three semicircular canals oriented in the three dimensions of space, and a saccule and utricle that detect linear acceleration and gravity. The inner ear contains six sensory epithelia, which contain the sensory hair cells. Vestibular hair cells in the two maculae and three cristae detect motion of the head, whereas auditory hair cells in the organ of Corti respond to specific frequencies of sound. Vestibular and auditory information is transmitted from the inner ear to the brain by primary sensory neurons in the vestibular or spiral ganglia respectively.

The inner ear provides an unusually sensitive system for analysis of gene function since small changes in the formation or structure of the inner ear can cause profound functional deficits in hearing and balance. For instance, *Lrig3* mutant mice exhibit hyperactivity and run in circles due to truncation of a single semicircular canal [28]. Further, Lrigs have been shown to modulate BMP, FGF, and Wnt signaling pathways, which all play important roles in the morphogenesis and patterning of the inner ear [35]. Thus, analysis of the inner ear provides an ideal opportunity to uncover the *in vivo* actions of the Lrigs.

Here, we analyzed several features of inner ear development and function in single and double *Lrig* mouse mutants. Our results suggest that *Lrig1* and *Lrig3* cooperate during morphogenesis. *Lrig1* and *Lrig2*, on the other hand, control largely distinct aspects of inner ear development and function, yet act redundantly to ensure proper innervation of the cochlea.

## Results

To be able to compare and contrast *Lrig* gene function in the inner ear, it is critical to know when and where each family member is expressed. Any sites of overlap offer an opportunity to examine redundancy, whereas unique sites of expression can be used to reveal the biological significance of individual family members. For instance, *Lrig3* is the only family member expressed in the developing lateral semicircular canal and *Lrig3* mutant mice circle due to defects in this canal. However, although *Lrig3* is also expressed in other regions of the inner ear, *Lrig3* mutant mice exhibit normal auditory responses, with no other obvious changes in the structure or function of the inner ear [28]. This raises the possibility that other *Lrig* genes compensate for the loss of *Lrig3*. Therefore, to begin to determine whether these three family members play overlapping functions, we compared their expression patterns in the inner ear, either by *in situ* hybridization (*Lrig1*) or by examining the expression of β*geo* reporter genes inserted into the *Lrig2* (Figure S1) and *Lrig3* [28] loci.

**Figure 1:**
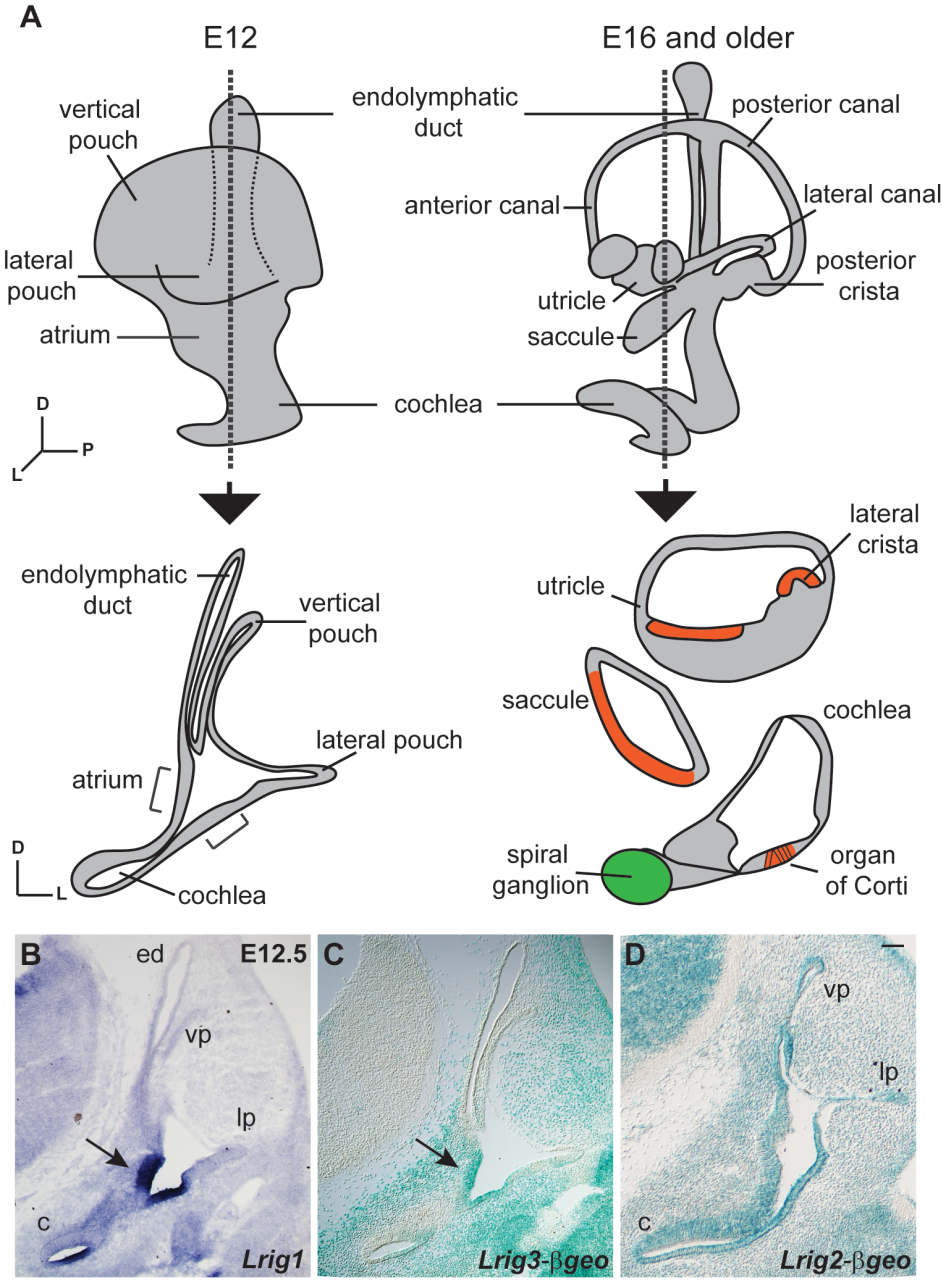
Lrigs are co-expressed in the embryonic inner ear. (A) Diagram of the immature inner ear structure at E12.5 (left) and the mature structure at E16 (right), with schematic cross sections cut transverse to the ear at each age shown below. For the E16 cross section, the sensory epithelia are labeled red and the neurons green. *In situs* for *Lrig1* (B) and X-gal staining for Lrig3-βgeo activity (C) show overlapping expression for *Lrig1* and *Lrig3* in the atrium (arrowhead) and the non-sensory domain of the cochlea. On the other hand, Lrig2-βgeo is active throughout the developing otic epithelium (D). c = cochlea, ed = endolymphatic duct, lp = lateral pouch, vp = vertical pouch. Scale bar = 50 µm.

Given the known role for *Lrig3* in canal morphogenesis, we first compared expression patterns at embryonic day 12.5 (E12.5), just before the canals begin to acquire their mature morphology. The inner ear develops from the otic vesicle, a simple sphere of epithelium that invaginates from the epidermis overlying the hindbrain beginning around E9 in mouse [35]. Over the next several days, the vestibular apparatus and endolymphatic duct develop from the dorsal half of the otic vesicle, while the cochlea extends ventrally (Figure 1A). Beginning around E12, the semicircular canals are sculpted from the vertical and lateral pouches. The utricle and saccule develop from an intermediate region called the atrium [37]. In parallel, signaling events establish restricted sensory regions, which ultimately produce hair cells and support cells in the mature sensory epithelia in the canals (the cristae), the utricle and saccule (the maculae), and the cochlea (the organ of Corti). Non-sensory regions in the cochlea go on to form the lateral wall, inner sulcus, and Reissner's membrane. Consistent with previous studies [28], *Lrig1* and *Lrig3* showed remarkably restricted yet related patterns of expression at E12.5, overlapping both in the atrium and in the non-sensory domain of the cochlea (Figure 1B, C). In contrast, Lrig2-βgeo activity was evident throughout the early otic epithelium (Figures 1C and S2). Indeed, Lrig2-βgeo expression appeared nearly ubiquitous at all stages examined, although the levels varied in different tissues (Figure S2).

**Figure 2:**
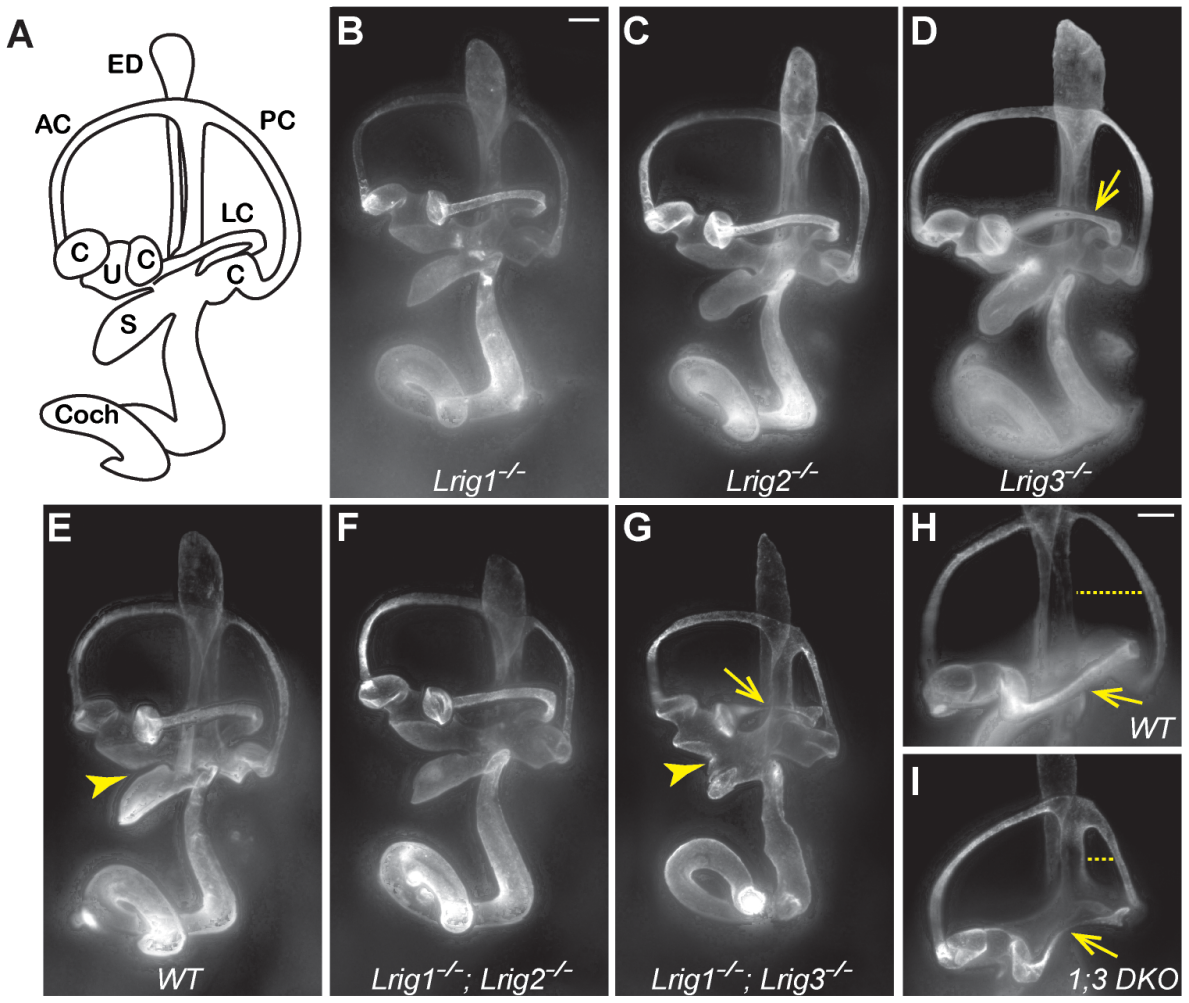
Lrig1 and Lrig3 act redundantly during inner ear morphogenesis. (A) Schematic of the mouse inner ear. (B–I) Light microscopy images of E14.5 inner ears filled with paint. *Lrig1^−/−^* (B), *Lrig2^−/−^* (C), and *Lrig1^−/−^;Lrig2^−/−^* double mutants (F) exhibit no changes in inner ear morphology when compared to wild-type controls (E). On this genetic background, loss of both copies of *Lrig3* causes a partially penetrant thinning or truncation of the lateral canal (arrows, D, G, I). Interestingly, *Lrig1^−/−^;Lrig3^−/−^* double mutants present additional morphogenetic phenotypes including a failure of the utricle and the saccule to separate (compare arrowheads in E and G) and a smaller misshapen posterior canal (compare dashed line in H and I). AC = anterior semicircular canal, C = crista, Coch = cochlea, ED = endolymphatic duct, LC = lateral semicircular canal, PC = posterior semicircular canal, S = saccule, U = utricle. Scale bar = 100 µm.

To determine whether *Lrig1* and *Lrig2*, like *Lrig3*, help determine the three-dimensional structure of the inner ear, we generated and analyzed *Lrig1* and *Lrig2* mutant mice. *Lrig1* mutant mice harbor a gene trap insertion in the third intron of the *Lrig1* locus, and *Lrig2* mutants contain a gene trap insertion after exon 11 (Figure S1). These gene trap insertions are predicted to interfere with normal splicing of endogenous transcripts, instead producing transmembrane fusion proteins that are targeted to the lysosome and therefore unlikely to exert any effect [38]. Western blot and immunostaining studies confirmed that Lrig1 and Lrig2 protein levels are severely reduced in each mutant background (Figures S1 and S3). In contrast to *Lrig3* mutants, however, both *Lrig1* and *Lrig2* single mutant animals exhibited normal inner ear morphologies at E14.5 (Fig. 2B, C, E).

Given the striking co-expression of *Lrig1* and *Lrig3*, we wondered whether combined loss of these two family members would provide any evidence for similar functions. Indeed, inner ear development is more severely disrupted in *Lrig1^−/−^;Lrig3^−/−^* double mutant mice than in either single mutant (Table 1). For example, the utricle and saccule fail to separate (Figure 2G, arrowhead), consistent with the co-expression of *Lrig1* and *Lrig3* in the embryonic atrium (Figure 1). In addition, the posterior canal is abnormally small and misshapen (Figure 2H, I). To see whether *Lrig1* and *Lrig3* also cooperate in the lateral canal, we took advantage of the fact that the lateral canal phenotype is only partially penetrant in *Lrig3* mutants maintained on this background, with truncation or thinning observed in only 33% of the animals (Figure 2D, Table 1). However, loss of either one or two copies of *Lrig1* did not strongly enhance this phenotype (Figure 2D, G, I; Table 1), consistent with the fact that *Lrig1* and *Lrig3* are not obviously co-expressed in the lateral canal epithelium [28]. The fact that new phenotypes emerge only in sites of *Lrig1/Lrig3* co-expression strongly suggests that these two family members act redundantly during inner ear morphogenesis. In contrast, *Lrig1* and *Lrig2* do not appear to cooperate here, as *Lrig1^−/−^;Lrig2^−/−^* double mutant ears developed normally (Figure 2F) despite the extensive co-expression of *Lrig1* and *Lrig2* at E12.5 (Figure 1B, D).

**Table 1:** Paintfill analysis reveals inner ear morphological defects in *Lrig* mutants.

*Lrig1; Lrig3* genotype	n =	Lateral canal defect	Posterior canal defect	Saccule/utricle defect
***+/+; +/+***	8	0	0	0
***+/−; +/−***	9	0	0	0
***−/−; +/+***	7	0	0	0
***−/−; +/−***	10	0	0	0
***+/+; −/−***	12	4 (33%)	0	0
***+/−; −/−***	10	2 (20%)	0	0
***−/−; −/−***	10	5 (50%)	6 (60%)	10 (100%)

Inner ear morphology was assessed blind to genotype in animals with all possible combinations of *Lrig1* and *Lrig3* mutant alleles. “n” corresponds to the total number of ears that were scored for each genotype. Columns indicate the number of ears of each genotype that showed defects in the lateral canal, posterior canal, or saccule/utricle, with the percent of total ears examined in parentheses. Novel phenotypes were observed only in *Lrig1^−/−^;Lrig3^−/−^* double mutant animals.

To gain a broader view of genetic interactions among *Lrig* family members, we asked whether either *Lrig2* or *Lrig3* exert overlapping functions with *Lrig1* in other regions of the inner ear. In support of this idea, unlike either single mutant, *Lrig1^−/−^; Lrig3^−/−^* double mutant animals die at or before birth (Table 2) and suffer from an array of morphogenetic phenotypes, including microphthalmia and skeletal malformations (data not shown). Although the presence of new defects suggests that *Lrig1* and *Lrig3* likely work together in many other tissues, this lethality prevented analysis of any other aspects of inner ear function. In contrast, *Lrig1^−/−^*, *Lrig2^−/−^*, and *Lrig1^−/−^;Lrig2^−/−^* mutant mice survive past the onset of hearing. We therefore focused the rest of the analysis on *Lrig1* and *Lrig2*.

**Table 2:** Genotype distribution of *Lrig* mutant animals.

*Lrig1* ^+/−^;*Lrig2* ^+/−^×*Lrig1* ^+/−^;*Lrig2* ^+/−^ intercross, 253 mice genotyped. χ^2^ p-value: <0.0001 at 6 weeks									
*Lrig1*	+/+	+/+	+/−	+/−	+/+	+/−	−/−	−/−	−/−
*Lrig2*	+/+	+/−	+/+	+/−	−/−	−/−	+/+	+/−	−/−
Observed and (expected) at P7	22 (16)	30 (32)	37 (32)	66 (63)	20 (16)	43 (32)	11 (16)	18 (32)	6 (16)
Observed at 6 weeks	22	30	37	66	20	40	8	13	2

*Lrig1* ^+/−^;*Lrig3* ^+/−^×*Lrig1* ^+/−^;*Lrig3* ^+/−^ intercross, 181 mice genotyped. χ^2^ p-value: <0.025 at 6 weeks									
*Lrig1*	+/+	+/+	+/−	+/−	+/+	+/−	−/−	−/−	−/−
*Lrig3*	+/+	+/−	+/+	+/−	−/−	−/−	+/+	+/−	−/−
Observed and (expected) at P7	14 (11)	28 (23)	24 (23)	45 (45)	11 (11)	22 (23)	12 (11)	19 (23)	0 (11)
Observed at 6 weeks	14	28	24	45	11	21	9	14	0

Mice were generated from intercrosses between animals carrying *Lrig1* and *Lrig2* (top) or *Lrig1* and *Lrig3* mutant alleles (top). The number of mice carrying each genotype at postnatal day 7 (P7) and at 6 weeks is indicated, out of a total of 253 offspring from *Lrig1*;*Lrig2* intercrosses and 181 offspring from *Lrig1*;*Lrig3* intercrosses. For each genotype, the number of mice that is expected from this kind of intercross in indicated in parentheses. χ-squared tests confirmed that the observed distribution of genotypes is significantly different from the expected distribution.

As previously reported [20], *Lrig1^−/−^* mice frequently die within the first postnatal week when maintained on an inbred background (Table 2). *Lrig2^−/−^* mice were born in normal Mendelian ratios and showed no obvious defects (Table 2). However, very few *Lrig1^−/−^;Lrig2^−/−^* double mutant animals survived to six weeks of age. A small percentage of *Lrig1^−/−^; Lrig2^−/−^* double mutants survived to adulthood (Table 2) and were noticeably runty during adolescence. More strikingly, half of the double mutant survivors exhibited a mild vestibular defect with circling behavior (3 of 6 animals, see video S1). Since neither surviving *Lrig1^−/−^* nor *Lrig2^−/−^* animals showed any signs of circling, this observation suggests that Lrig1 and Lrig2 may work together in the vestibular system.

To investigate this possibility, we performed a more detailed analysis of expression in the vestibular system by double labeling with an anti-Lrig1 antibody and an anti-β-galactosidase antibody to detect Lrig2-βgeo. *Lrig2* gene trap heterozygotes were used due to the lack of antibodies that reliably detect Lrig2 protein in tissue. Lrig2-βgeo should serve as an accurate read-out of the pattern of *Lrig2* expression, but it should be noted that there may be subtle differences in the stability of the Lrig2-βgeo protein compared to endogenous Lrig2. However, our observations of Lrig2-βgeo expression match previous reports of *Lrig2* transcription [10], so any discrepancies are likely to be minor. No Lrig1 labeling was detected in *Lrig1* mutant tissue, confirming that this antibody detects only this family member (Figure S3).

**Figure 3:**
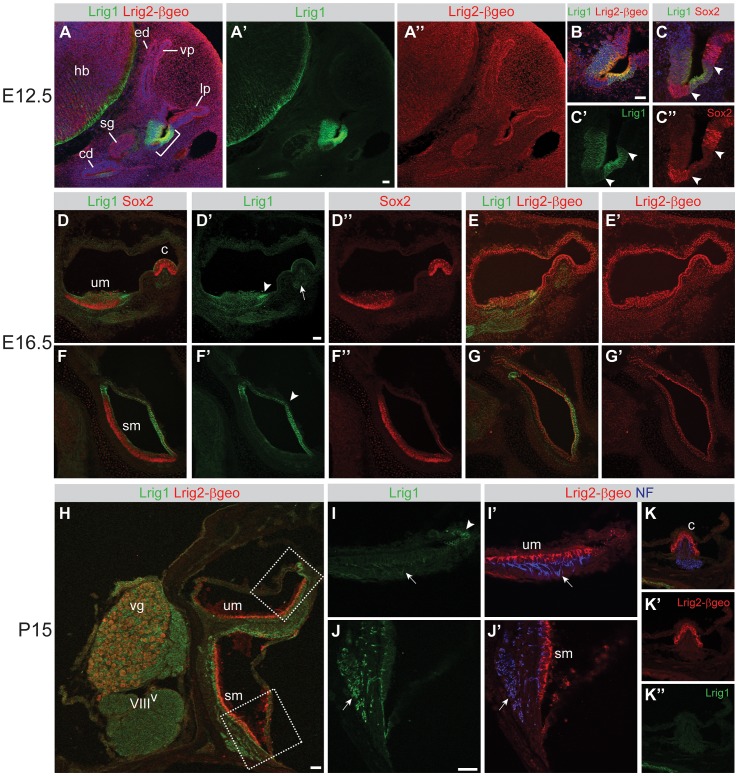
Lrig1 and Lrig2-βgeo are co-expressed in non-sensory tissues and in the vestibular ganglion. Transverse sections through *Lrig2^+/−^* tissue at E12.5 (A–C), E16.5 (D–G), and P15 (H–K) were double labeled with combinations of antibodies to Lrig1, β-galactosidase (to detect Lrig2-βgeo), Sox2, and neurofilament (NF). (A) At E12.5, Lrig1 was detected in the atrium (bracket), while Lrig2-βgeo was present throughout the otic epithelium and surrounding mesenchyme (A″), overlapping with Lrig1 in the atrium (B). Within the atrium, Lrig1 was present in non-sensory tissues that flank Sox-2 positive sensory regions (arrowheads, C–C″). This expression was maintained at E16.5, with Lrig1 in the transitional epithelium adjacent to the utricular macula (arrowhead, D′) and in the extramacular epithelium of the saccule (arrowhead, F′), as well as in vestibular projections to the utricle and lateral crista (arrow, D′). Lrig2-βgeo, on the other hand, continued to be expressed broadly in both sensory and non-sensory portions of the vestibular organs at E16.5 (E′, G′). After the onset of hearing (P15), Lrig1 was expressed in NF-positive fibers innervating the utricular and saccular maculae (arrows, I, J), whereas Lrig2-βgeo was enriched in all vestibular sensory epithelia (I′,J′,K), which were recognized by the presence of NF labeled projections. c = crista, cd = cochlear duct, ed = endolymphatic duct, hb = hindbrain, lp = lateral pouch, sg = spiral ganglion, sm = saccular macula, um = utricular macule, vg = vestibular ganglion, vp = vertical pouch, VIII^V^ = vestibular division of the eighth cranial nerve. Scale bar = 40 µm.

Consistent with results from *in situ* hybridization and X-gal staining (Figures 1 and S2), double labeling at E12.5 revealed highly restricted expression of Lrig1 protein in the atrium and cochlea, with broad Lrig2-βgeo expression throughout the otic epithelium and in the surrounding mesenchyme (Figure 3A, B). Within the atrium, Lrig1 was restricted to non-sensory regions, which flank the Sox2-positive sensory patches that eventually give rise to the maculae (Figure 3C). This pattern was maintained after formation of the utricle and saccule, with expression in the transitional epithelium adjacent to the utricular macula and in the extramacular epithelium of the saccule at E16.5 (Figure 3D, F), E18.5 (Figure S3) and P15 (Figure 3H–J). Lrig2-βgeo, in contrast, was expressed throughout sensory and non-sensory regions of the vestibular organs at E16.5 and continuing through the first postnatal week (Figure 3E, G and S2). However, by P15, Lrig2-βgeo levels were noticeably enhanced in the utricular and saccular maculae as well as the cristae (Figure 3E, G and data not shown). In contrast, Lrig1 protein was not detected in the vestibular sensory epithelia at any stage. Thus, Lrig1 and Lrig2 are co-expressed in non-sensory regions of the utricle and saccule, but only Lrig2 seems to be present in the sensory epithelia.

One prominent site of overlapping expression was the vestibular ganglion, which communicates head position information to the brain. Lrig1 was present at low levels in the neuronal cell bodies, with intense expression in projections to the utricle, saccule, and lateral crista at E16.5 (Figure 3D), E18.5 (Figure S3), and P15 (Figure 3H–J). Lrig2-βgeo was also present in the vestibular ganglion at all stages, with enriched expression at P15 (Figures 3H and S2, and data not shown). The co-expression of *Lrig1* and *Lrig2* in the vestibular ganglion, particularly at postnatal stages, may explain why *Lrig1^−/−^;Lrig2^−/−^* double mutant animals display occasional circling behavior, since the gross structure of the inner ear is unaffected (Figure 2F) and *Lrig1* and *Lrig2* are not co-expressed in the sensory epithelia at any stage (Figure 3). Thus, it is possible that *Lrig1* and *Lrig2* act redundantly in the vestibular ganglion neurons or non-sensory epithelium, though they do not cooperate during the initial formation of the vestibular apparatus.

**Figure 4:**
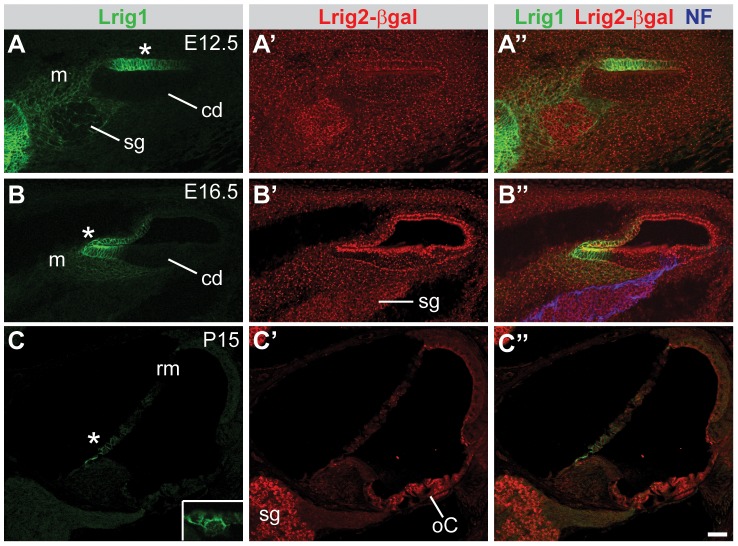
Lrig1 and Lrig2-βgeo are co-expressed in the non-sensory region of the cochlea. Transverse sections through *Lrig2^+/−^* tissue at E12.5 (A), E16.5 (B), and P15 (C) were double labeled with antibodies to Lrig1, β-galactosidase, and NF. (A) At E12.5, staining was evident in the non-sensory region of the cochlear epithelium (asterisk) and the mesenchyme surrounding the spiral ganglion. (B) At E16.5, Lrig1 was detected in the medial wall of the cochlea, which will form the inner sulcus and Reissner's membrane (asterisk). (C) At P15, Lrig1 was found in the base of Reissner's membrane (asterisk), with localization to the cell surface (inset). In contrast, at E12.5 and 16.5, Lrig2-βgeo was found broadly in the cochlear epithelium and surrounding mesenchyme (A′–B′). At P15, expression was enriched in spiral ganglion neurons and in the organ of Corti (C′). cd = cochlear duct, m = mesenchyme, oC = organ of Corti, rm = Reissner's membrane, sg = spiral ganglion. Scale bar = 40 µm.

As in the vestibular system, Lrig1 and Lrig2 showed largely distinct patterns of expression in the cochlea, overlapping only in non-sensory regions. Lrig1 protein was restricted to non-sensory regions of the cochlea at all stages, with maintained expression only in Reissner's membrane, a structure that regulates the endolymph environment that is critical for cochlear function (Figure 4A–C) [39]. Lrig2-βgeo, on the other hand, appeared ubiquitous in the cochlear epithelium and surrounding mesenchyme at E12.5 and E16.5 (Figure 4A′–B′). However, similar to the vestibular system, expression was elevated in sensory and neural tissues postnatally (Figures 4C′ and S2). Although Lrig1 was not detected in the spiral ganglion neurons or their projections at any stage, expression was apparent in the mesenchyme in the region that the spiral ganglion neuron neurites grow through to reach the cochlear duct (Figure 4A, and data not shown). In summary, although *Lrig1* and *Lrig2* are at times co-expressed in the vestibular system and cochlea, these two family members show fundamentally different expression patterns, which contrasts with the obvious similarities in the expression of *Lrig1* and *Lrig3* at all stages examined (Figure 1 and [28]).

To assess the relative contributions of *Lrig1* and *Lrig2* to cochlear function, we tested auditory responsiveness in single and double mutant mice using two complementary assays. First, we recorded Distortion Product Otoacoustic Emissions (DPOAEs), which are generated by the cochlea in response to simultaneous presentation of two slightly dissimilar pure tone frequency stimuli. Production of DPOAEs depends on outer hair cell (OHC) function, and DPOAE thresholds will increase if hair cells are missing, damaged, or cannot be properly stimulated due to changes in cochlear mechanics. Second, we recorded Auditory Brainstem Responses (ABRs). ABRs reflect the sum of neuronal activity in response to sound stimulation, starting with the initial activation of spiral ganglion neurons (wave 1) and following with activation in the auditory brainstem (waves 2–5). Sensitivity is assessed by determining the lowest intensity sound stimulus (i.e. the threshold) that is able to generate an ABR response. In addition, the strength of the neuronal response can be evaluated by measuring the latency and amplitude of the first wave. By altering the frequency of the pure tone stimuli, function can be tested along the length of the cochlea, from high frequencies in the base to low frequencies in the apex. Together, these tests offer a sensitive way to identify impairments in the ability of the cochlea to detect and respond to acoustic stimuli.

**Figure 5:**
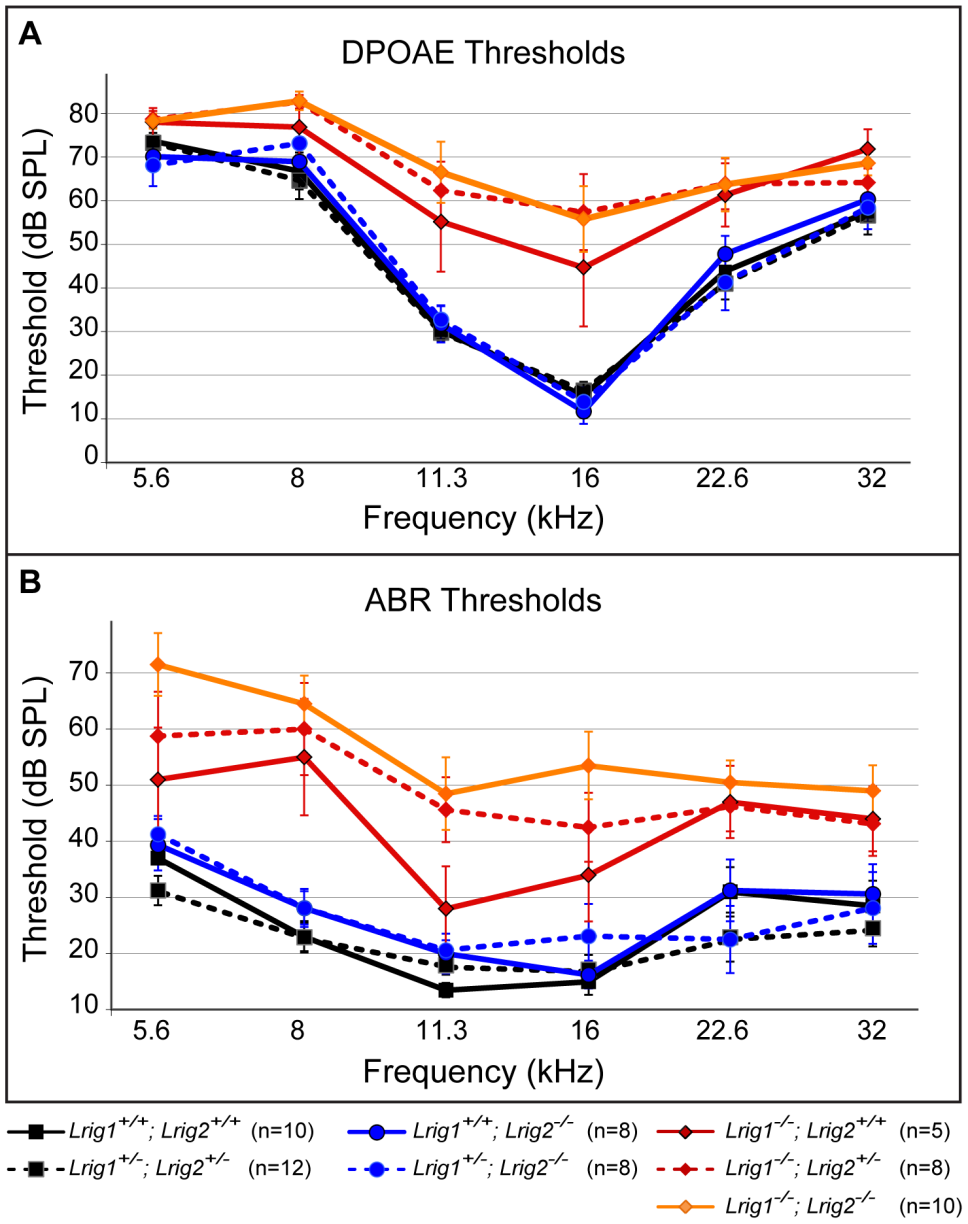
*Lrig1* but not *Lrig2* mutant mice exhibit decreased auditory sensitivity. Plots of threshold values from DPOAE recordings (A) and ABRs (B) performed on 6 week old animals. Auditory responses were tested at six frequencies (from low (5.6 kHz) to high (32 kHz)), and across a range of intensities (from sound pressure levels of 10 to 80 decibels (dB)). DPOAE and ABR thresholds in *Lrig2* mutant animals (solid blue line) were normal when compared to control animals (solid black line), even when additionally heterozygous for *Lrig1* (dashed blue line). In contrast, *Lrig1^−/−^* mutants (solid red line) showed a moderate increase in both DPOAE and ABR thresholds. This effect was even stronger in *Lrig1^−/−^;Lrig2^+/−^* mutant animals (dashed red line) and *Lrig1^−/−^;Lrig2^−/−^* (solid orange line), which experienced a severe decrease in sensitivity across all frequencies. See Tables S1 and S2 for raw data and analysis of statistical significance.

DPOAE and ABR measurements revealed that *Lrig1*, but not *Lrig2*, is necessary for normal auditory sensitivity. *Lrig1* mutants showed significantly elevated DPOAE and ABR thresholds in response to 11.3 and 16 kHz stimuli, which typically elicit the lowest threshold responses in control animals (Figure 5 and Table S2). Whereas control animals reliably detected 16 kHz DPOAE stimuli as quiet as 15 dB, mutants did not respond until the sounds were 45 dB, which is ∼30 times more intense. Thresholds were also elevated in response to lower (5.6 and 8 kHz) and higher (22.6 and 32 kHz) frequencies, but these differences were not statistically significant since sensitivity is already reduced in these regions of control cochleae (for example, 57.43±2.37 dB for wild-type vs. 71.86±4.5 dB for *Lrig1^−/^* ^−^ animals presented with a 32 kHz stimulus). *Lrig2* mutants, on the other hand, responded with the same sensitivity as control littermates. Similarly, *Lrig1^+/−^*;*Lrig2^−/−^* mutants also demonstrated normal thresholds. However, loss of either one or two copies of *Lrig2* from *Lrig1* mutants strongly enhanced the effect, such that the outer hair cell response of *Lrig1^−/−^;Lrig2^+/−^* and *Lrig1^−/−^;Lrig2^−/−^* animals only occurred in response to sounds greater than 55 dB across all frequencies (Table S1).

**Figure 6:**
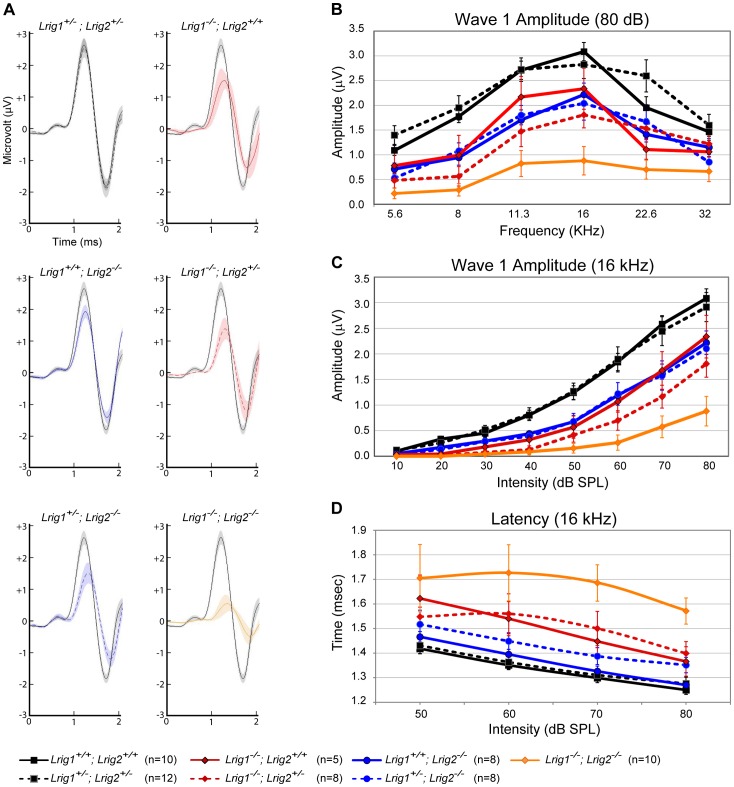
ABR amplitudes are reduced in both *Lrig1* and *Lrig2* mutant mice, and this effect is enhanced in double mutants. (A) For each genotype analyzed, ABR waveforms for the first wave were averaged and overlaid with an average wild-type waveform (solid black line). The shading indicates the standard error of the mean. The first wave is reduced in *Lrig1* and *Lrig2* single mutants, as well as in animals mutant for three out of four alleles (*Lrig1^−/−^;Lrig2^+/−^* and *Lrig1^+/−^;Lrig2^−/−^*). This phenotype is enhanced in *Lrig1^−/−^;Lrig2^−/−^* mutant animals, which show a much reduced response. (B) Quantification of the diminished ABR response for all genotypes. Plots show the amplitudes of the first wave in response to six different pure tone stimuli each presented at 80 dB. (C) Plot of wave 1 amplitudes in response to 16 kHz pure tone stimuli presented from quiet (10 dB) to loud (80 dB) intensities. Mutant mice responded worse to the stimulus even at low sound pressure levels. (D) Plot of the latency of wave 1 in response to a 16 kHz stimulus presented at four different sound intensities. The response is significantly delayed in double mutant animals. See Tables S3 and S4 for raw data and analysis of statistical significance.

To understand how loss of *Lrig2* might exacerbate the *Lrig1* phenotype, we looked more closely at the nature of the ABR waveforms in all single and double mutant combinations (Figure 6). As expected, in *Lrig1* mutants the amplitude of the first wave was significantly diminished in response to a range of frequencies and sound intensities (Figure 6C, D, and Tables S3 and S4). Combined with the increased thresholds, this suggests that the neural response is decreased because the cochlea is not able to detect sounds with sufficient sensitivity. Remarkably, despite the lack of any effect on thresholds, *Lrig2* mutants showed a similar response: the amplitude of the first wave was significantly decreased relative to controls at multiple frequencies and across sound intensities (Figure 6C, D, and Tables S3 and S4). Latencies were also increased (Figure 6D). Thus, whereas *Lrig1* is critical for the initial detection of sound, *Lrig2* is required for the subsequent neuronal response. Since Lrig2 is uniquely enriched in the spiral ganglion neurons throughout life, these findings suggest that Lrig1 and Lrig2 control distinct aspects of cochlear function. Amplitudes and latencies were even more affected in double mutants, as expected based on the increased thresholds.

**Figure 7:**
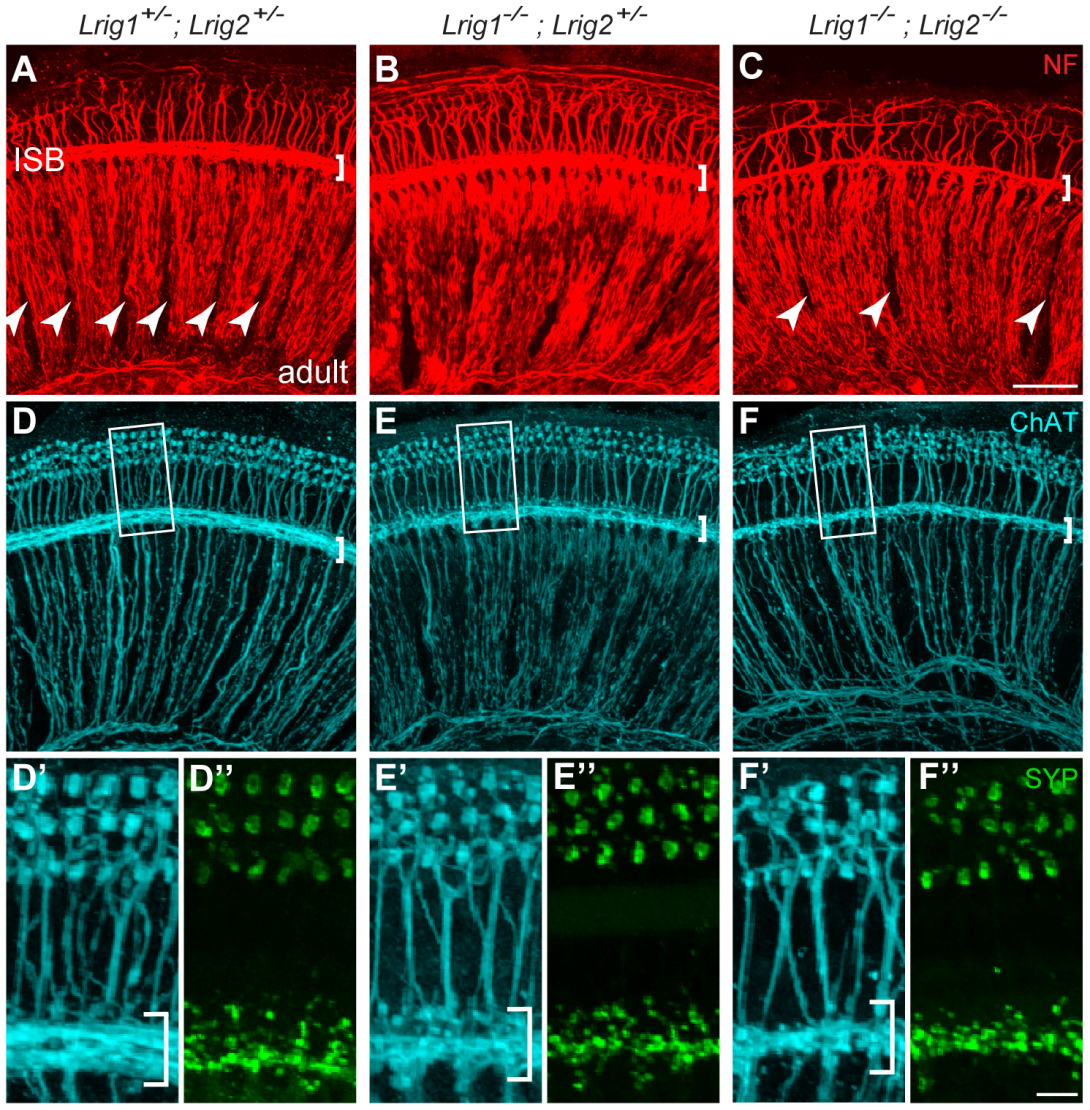
Cochlear structure and patterning is normal in *Lrig1^−/−^*;*Lrig2^−/−^* double mutants but cochlear innervation is disrupted. (A–C) Cochlear tissue from adult animals was immunostained and then imaged as a flat-mount by confocal microscopy. In controls (A), neurofilament (NF)-positive neurites from both afferent and efferent neurons align in distinct radial bundles. Efferent projections also travel non-radially along the length of the cochlea in the inner spiral bundle (ISB) (bracket in A). The regular spacing normally found between these axonal bundles (A, arrowheads) is disrupted in *Lrig1^−/−^*;*Lrig2^−/−^* double mutants (C, arrowheads). In addition, the ISB is reduced (brackets). Innervation was grossly normal in *Lrig1^−/−^;Lrig2^+/−^* animals. (D–F) Efferent axons and their terminals were visualized by staining for Choline acetyltransferase (ChAT) (D–F and D′–F′) and synaptophysin (Syp) (D″–F″). High power images of the boxed regions illustrate the obvious reduction in efferent innervation in double mutants (F′, F″) compared to controls (D′, D″). *Lrig1^−/−^;Lrig2^+/−^* animals showed an intermediate effect (E′, E″). ISB = inner spiral bundle, oC = organ of Corti, sg = spiral ganglion. See Figure S4 for images of additional genotypes. (A–F) Scale bar = 50 µm. (D′–F″) Scale bar = 10 µm.

Although *Lrig1^−/−^;Lrig2^−/−^* double mutants exhibit a fully penetrant auditory response deficit, the cochlea showed no gross malformations either at E19 (Figure S4A, B) or in adults (data not shown). The cochlear duct had a normal histological appearance, consistent with the absence of any morphological defect at E14.5 (Figure 2). In addition, immunostaining confirmed the presence of hair cells and neurons in each turn of the cochlea, with spiral ganglion neurites extending to contact hair cells in the organ of Corti (Figure S4C, D). Similarly, in the few double mutant animals that survived past early postnatal stages, there was no obvious change in the number or organization of hair cells and spiral ganglion neurons (data not shown). However, the overall pattern of cochlear innervation was clearly disrupted in double mutants, as revealed by immunolabeling for neurofilament, which labels both afferent and efferent neurites (Figure 7A–C). Whereas control neurites aligned in regularly spaced radial bundles that were clearly separated from each other (Figure 7A), the mutant neurites were noticeably defasciculated and the gaps between the bundles were smaller and present only intermittently (Figure 7C). More strikingly, the inner spiral bundle (ISB, bracket) was reduced, indicating a possible change in the innervation of the cochlea by efferent neurons from the hindbrain. In contrast, no obvious changes were apparent in *Lrig1* or *Lrig2* single mutants (Figure S4E–G).

To determine whether *Lrig1* and *Lrig2* might act redundantly in certain contexts, we looked more closely at the efferent innervation of the cochlea by staining for choline acetyltransferase (ChAT) [40] and synaptophysin in single and double mutant animals. Consistent with results from neurofilament-staining, efferent innervation of the cochlea was noticeably sparser in double mutant animals (n = 4) compared to controls (n = 8) (Figure 7D, F). In contrast, cochleae from *Lrig1^−/−^* (n = 2) and *Lrig1^+/−^;Lrig2^−/−^* (n = 4) animals were unaffected (Figure S4G, H). Due to the nature of the crosses used to generate sufficient numbers of double mutant animals, *Lrig2^−/−^* single mutant animals were not available for analysis of efferent innervation. However, the normal pattern of neurofilament staining (Figure S4G) together with the lack of defects in the *Lrig1^+/−^;Lrig2^−/−^* cochlea (Figure S4H′) indicates that *Lrig2* is not required on its own and that Lrig1 can fully compensate for reduced Lrig2 activity. On the other hand, cochleae from *Lrig1^−/−^;Lrig2^+/−^* animals (n = 4) exhibited an intermediate phenotype (Figure 7E), which fits with their diminished auditory responsiveness. Taken together, these findings indicate that *Lrig1* and *Lrig2* exert overlapping functions during cochlear innervation, perhaps uncovering a novel role for Lrig proteins in the nervous system. Moreover, the absence of any obvious morphogenetic or gross cochlear patterning defects argues against the idea that Lrig1 and Lrig2 act redundantly to control any of the major signaling pathways, consistent with their distinct effects *in vitro* and in cancer.

## Discussion

Here, we used genetic analysis in mice to compare and contrast the effects of *Lrig2* and *Lrig3* to the founding member of the family, *Lrig1*. By analyzing multiple aspects of inner ear development and function, we found that *Lrig1* and *Lrig3* cooperate to control inner ear morphogenesis, whereas *Lrig1* and *Lrig2* appear to affect largely distinct aspects of inner ear function. Our results highlight the biological significance of all three *Lrig* genes *in vivo* and provide insights into the functional diversity of the LRR-Ig superfamily of proteins.

Our findings add to a growing body of work underscoring the similarities between Lrig1 and Lrig3. At the molecular level, both Lrig1 and Lrig3 can bind multiple members of the EGF receptor family and show a similar subcellular distribution, with expression on the cell surface and in intracellular vesicles [25], [41]. Moreover, both family members also interact with other rTKs [23], [24], [27], indicating that the Lrig ectodomain does not mediate selective binding. In addition, *in vitro* studies suggest that both Lrig1 and Lrig3 can act as negative regulators of signaling pathways [16], [17], [23], [24], [26], [27]. Our findings suggest that Lrig1 and Lrig3 also exhibit common activities *in vivo*. For instance, *Lrig1* and *Lrig3* show strikingly similar patterns of expression within multiple tissues throughout development [10], [28]. Moreover, *Lrig1^−/−^;Lrig3^−/−^* double mutants exhibit much more dramatic phenotypes than either single mutant. Importantly, new phenotypes emerge at sites of co-expression, such as the developing utricle and saccule. Conversely, the strongest phenotype in the *Lrig3* mutant ear is in the lateral canal, which is one of the few sites where *Lrig1* and *Lrig3* do not overlap.

Curiously, although Netrin1 is a key effector of Lrig3 activity in the lateral canal, the atrium develops normally in *Netrin1^−/−^* mice (A.M.N. and L.V.G., unpublished observation), suggesting that Lrig1 and Lrig3 mediate their effects through additional molecules in this region of the inner ear. Consistent with this idea, neither the anterior nor posterior canal was truncated in *Lrig1^−/−^;Lrig3^−/−^* double mutant inner ears, despite the known role of Netrin1 there [42]. One likely explanation is that Lrig1 and Lrig3 modulate a broadly active signaling pathway that controls expression of *Netrin1* in the lateral canal, but that other target genes are responsible for effects elsewhere in the inner ear. Indeed, our results suggest that both of these Lrig proteins mediate their effects through key signaling pathways underlying morphogenesis, as *Lrig1^−/−^;Lrig3^−/−^* double mutants die at or before birth with obvious morphogenetic malformations in multiple tissues. A much more detailed analysis of each affected tissue will be needed to pinpoint the pathways involved.

Although Lrig1 and Lrig3 appear to cooperate during inner ear morphogenesis, each protein also has its own distinct biological functions. Indeed, the phenotypes already reported in *Lrig1* mutant mice indicate that this family member may play a particularly prominent role in EGF signaling and cell proliferation [20]–[22]. Similarly, despite the extensive overlap of *Lrig1* and *Lrig3* in the ear, loss of *Lrig1* is sufficient to cause a significant auditory phenotype, as evidenced by increased DPOAE and ABR thresholds. No auditory phenotypes were detected in *Lrig3* mutant mice, in contrast [28]. Moreover, a role for ErbB receptors in inner ear morphogenesis has not been described, and in fact, broad inhibition of ErbB activity has no effect on canal formation in chicks [25]. On the other hand, BMP and FGF signaling is critical for inner ear morphogenesis [35]. Thus, one possibility is that Lrig1 and Lrig3 work together to modulate signaling through BMP or FGF pathways, but that Lrig1 is the dominant regulator of the EGF pathway *in vivo*. In support of this idea, Lrig1 and Lrig3 can actually exert opposing effects on ErbB receptor levels *in vitro*, with Lrig1 reinforcing its effects by decreasing Lrig3 levels [18]. Hence, the added loss of *Lrig3* might not be expected to exacerbate the effects of *Lrig1* on EGF signaling *in vivo*. Whether Lrig1 and Lrig3 also exert reciprocal effects on the FGF receptor or other putative targets has not yet been examined. An important step towards resolving these apparent differences will be to determine the nature of the pathways affected by both Lrig1 and Lrig3 *in vivo*.

Analysis of *Lrig2* indicates that this family member has acquired particularly independent functions. Unlike *Lrig1* and *Lrig3*, *Lrig2* seems to be expressed nearly ubiquitously, although final confirmation awaits the production of reliable anti-Lrig2 antibodies. Such broad expression is not typical for proteins that function in developmental signaling pathways, which tend to show more restricted patterns of expression. Notably, despite the fact that *Lrig2* is apparently present in every site of *Lrig1* expression, no new morphogenetic phenotypes are uncovered in *Lrig1^−/−^;Lrig2^−/−^* double mutant mice. Thus, *Lrig2* is not sufficient to compensate for the combined loss of *Lrig1* and *Lrig3*, whereas *Lrig3* can direct proper inner ear morphogenesis even in the absence of both *Lrig1* and *Lrig2*. Although final proof will require analysis of triple mutant animals, the contrasting phenotypes seen in each set of double mutants strongly suggest that Lrig2 does not affect the same pathways as Lrig1 or Lrig3. These genetic results fit with previous reports that Lrig2 behaves differently *in vitro* and in human tumors [18], [29]–[31], [33]. It is also possible that some residual function persists in *Lrig2* gene trap mice, thereby obscuring redundant effects. However, this seems unlikely given the sensitivity of the inner ear to even subtle changes in signaling levels, as well as the fact that we were able to detect effects on auditory responsiveness in *Lrig2^−/−^* mutants. In addition, loss of just one copy of *Lrig2* was sufficient to exacerbate the *Lrig1^−/−^* phenotype, as assessed by DPOAE analysis. Although our work provides a useful starting point, analysis of independent *Lrig2* alleles may reveal additional functions for this protein in the future.

*Lrig2* seems to exert distinct effects from *Lrig1* and *Lrig3* on the basic signaling events that underlie patterning and morphogenesis, with an independent function in neurons. Indeed, although *Lrig2* mutant mice are outwardly normal, they do not process sound properly. Specifically, although mutant animals can detect sounds with normal sensitivity, the subsequent neuronal response is attenuated, as evidenced by significantly decreased ABR amplitudes across multiple frequencies. Consistent with this phenotype, *Lrig2* is present in spiral ganglion neurons throughout life, with particularly enhanced expression after the onset of hearing. How Lrig2 affects spiral ganglion neuron function remains unclear, though, as there were no obvious defects in the gross innervation of the cochlea in *Lrig2* mutant mice. This is not entirely unexpected, as many forms of human deafness are not associated with overt changes in the structure or organization of the cochlea. A role for Lrig2 in the brainstem may exist since *Lrig2* mutant mice exhibit auditory brainstem response deficits and *LRIG2* is crucial for brainstem mediated bladder control and facial expressions in humans [34]. Although hearing defects have not been reported, our findings suggest that it may be worth investigating whether any patients experience subtle auditory processing defects that might not be detected using standard auditory testing methods.

Our results also imply that *Lrig1* and *Lrig2* may cooperate in limited contexts. Indeed, *Lrig1^−/−^;Lrig2^−/−^* double mutant mice do show enhanced phenotypes relative to the single mutants. For instance, some double mutants show mild circling behavior and hyperactivity that is not seen in either single mutant. More strikingly, acoustic responsiveness is severely impaired in all double mutants, with both DPOAE and ABR thresholds increased across frequencies. The ABR effect may be mostly additive, as *Lrig1* and *Lrig2* single mutants each exhibit a different kind of auditory defect: *Lrig1* is required for the detection of sound and *Lrig2* is required for the appropriate neuronal response. The changes in DPOAE thresholds, on the other hand, could be due to redundancy, as loss of even one copy of *Lrig2* enhances the *Lrig1* mutant phenotype, despite the fact that thresholds are normal in *Lrig2* mutants. Since the amplification of the cochlear response by OHC activity is non-linear, DPOAEs offer an unusually sensitive measure of function. Hence, it is possible that the contribution of *Lrig2* is too minor to see on its own, but that this small effect is uncovered once OHCs stop responding optimally, as occurs in *Lrig1* mutants. Alternatively, *Lrig1* and *Lrig2* may play similar roles in Reissner's membrane, which is the only apparent site of co-expression in the mature cochlea. Reissner's membrane controls sodium homeostasis in the cochlear endolymph, and changes in the endolymph are known to lead to deafness [36], [39]. Intriguingly, *Lrig1* and *Lrig2* are also co-expressed in analogous non-sensory tissues in the utricle and saccule. Hence, a change in endolymph composition could also explain the mild vestibular phenotype uncovered in *Lrig1^−/−^;Lrig2^−/−^* double mutants.

An alternative explanation for the enhanced phenotypes is that Lrig1 and Lrig2 cooperate specifically in neuronal populations. In support of this idea, *Lrig1* and *Lrig2* are co-expressed in the vestibular ganglion and loss of both genes creates a vestibular deficit. More strikingly, efferent innervation is noticeably sparse in double mutant animals, but not obviously altered in either single mutant. The efferent neurons play an important role in modulating OHC responsiveness [43], so any change in their organization or function could cause the enhanced DPOAE phenotype. In support of this idea, an intermediate efferent phenotype was noted in *Lrig1^−/−^;Lrig2^+/−^* animals, paralleling their abnormal DPOAE responses. Understanding the origin of this defect will be challenging, however, due to the high lethality of *Lrig1^−/−^;Lrig2* ^−/−^ animals. Nevertheless, taken together with the proposal that the phenotypes seen in Urofacial Syndrome patients are due to abnormal innervation by neurons in the brainstem, these observations suggest that Lrig2 may play a particularly important role in the nervous system, much like the vertebrate-specific LRR-Ig proteins. Lrig1, on the other hand, may exhibit dual effects, acting like the ancient LRR-Ig proteins to regulate signaling in most contexts, but taking on new functions typical of other LRR-Ig proteins when present in neurons. Our findings underscore the need to delve more deeply into the functions of all three family members in the nervous system.

The divergence that occurs within the *Lrig* gene family may be analogous to the more general diversification in functions for the expanded LRR-Ig superfamily in vertebrates. Analysis of function across species strongly suggests that the original function of Lrig proteins is to bind rTKs and regulate their activity. There are single *Lrig* orthologs both in worms (*sma-10*) and flies (*lambik*). Although nothing is known about *lambik* function, *sma-10* is required for normal regulation of BMP signaling and hence body size in worms [44]. Interestingly, *lambik* can substitute for *sma-10 in vivo*. Similarly, Sma-10 binds both invertebrate and vertebrate BMP receptors. However, whereas Lrig1 acts as a negative regulator, *sma-10* has a positive effect on BMP signaling. Thus, Lrig proteins from diverse species appear to share the ability to bind to cell-surface receptors, but the consequences of these interactions vary. Similarly, Lrig family members within a single species may have diverged to acquire distinct signaling properties mediated by their intracellular domains. In the case of Lrig1 and Lrig3, the divergence from ancestral Lrig function is minimal. Lrig2, however, seems to have gained new and distinct functions. The presence of new behavioral phenotypes in *Lrig1^−/−^;Lrig2^−/−^* double mutant mice suggests that this poorly understood activity may in fact be shared by Lrig1 in some contexts. Although the view of *Lrig* function *in vivo* is far from complete, our findings may provide important insights into the origin and activities of vertebrate-specific branches of the LRR-Ig superfamily.

## Materials and Methods

### Mice

All mice were back-crossed and maintained for more than six generations on the C57BL/6N strain (Charles River Laboratories). The mouse line *Lrig1^Gt(GST4169C6)^* contains the VICTR48 gene trap vector (Lexicon Genetics) in the *Lrig1* locus (Figure S1) and was obtained from the Texas Institute for Genomic Medicine (TIGM) at Houston, TX via the Knock Out Mouse Program (KOMP) at the University of California, Davis. The *RST656* mouse line contains the GTOTMpfs gene trap vector [45] in the *Lrig2* locus (Figure S1). This results in production of a fusion between Lrig2 and βgeo, which mediates neomycin resistance as well as β-galactosidase activity all under the control of the endogenous *Lrig2* promoter. Mice were generated by the Mouse Gene Manipulation Facility of Boston Children's Hospital Intellectual and Developmental Disabilities Research Center (IDDRC) which is supported by NIHP30-HD18655. *Lrig3* mutant mice contain a deletion of exon 1 and were derived from the *Lrig3^flox^* allele which has been previously described [25]. Genotype distribution (Table 1) was assessed for surviving animals at 1 week and 6 weeks of age. For timed pregnancies, embryonic day 0.5 (E0.5) was defined as noon on the day a copulatory plug was present. All mice were maintained in accordance with institutional and National Institutes of Health (NIH) guidelines approved by the Institutional Animal Care and Use Committee (IACUC) at Harvard Medical School.

### Antisera production and Western blots

Rat polyclonal antiserum to Lrig2 was raised against the intracellular domain of mouse Lrig2 protein expressed in bacteria (Dana-Farber/Harvard Cancer Center Monoclonal Antibody Core). E12.5 littermate embryos were lysed in 50 mM Tris (pH 7.4), 150 mM NaCl, 1% Igepal CA 630 (NP-40), 0.5% sodium deoxycholate, 0.1% sodium dodecyl sulfate (SDS) and 1 mM Pefabloc (Roche). Western blot analysis was performed using standard protocols and a 1∶2000 dilution of anti-Lrig2 serum or 1∶8000 dilution of anti-actin antibody (Abcam ab8226).

### *In situ* hybridization

Non-radioactive *in situ* hybridization for *Lrig1* was performed on cryosections of mouse E12.5 tissue as described [28]. A detailed protocol is available at http://goodrich.med.harvard.edu/resources/resources_protocol.htm.

### X-gal staining

Tissue was fixed for 1 hour in 4% paraformaldehyde (PFA)/phosphate buffered saline (PBS), equilibrated in 30% sucrose/PBS at 4°C, and embedded in Neg50 (Richard-Allan Scientific). Cryosections transverse to the ear (Figure 1A) were cut and incubated in 1 mg/ml X-Gal (Sigma-Aldrich) in X-Gal buffer, post-fixed in 4% PFA/PBS for 1 hour at 4°C, and mounted using Glycerol Gelatin mounting medium (Sigma-Aldrich).

### Immunofluorescence

E12.5 and E16.5 mouse heads were collected and fixed for 1 hour at 4°C in 4% PFA/PBS, equilibrated in 30% sucrose/PBS at 4°C, and embedded in Neg50 (Richard-Allan Scientific). E19 heads were hemisected, fixed overnight at 4°C in 4% PFA/PBS and processed the same way. P15 animals were perfused with 4% PFA/PBS, the head hemisected and the brain removed, and the remaining tissue was post-fixed in 4% PFA/PBS for 1 hour at room temperature, decalcified in 0.12M EDTA/PBS overnight at room temperature followed by several days at 4°C, and embedded as before. Tissue from 6 week old animals was fixed overnight at 4°C in 4% PFA/PBS and decalcified for 5 to 7 days in 0.12M EDTA/PBS at 4°C prior to immunostaining. Cryosections cut transverse to the ear were blocked in PBS+3% bovine serum albumin (BSA) and permeabilized in wash solution (PBS+1% BSA+0.1% Triton X-100). Primary antibodies were added in wash solution at the following concentrations: β-galactosidase (1∶300, MP Biomedicals 08559761), Lrig1 (1∶75–300, R&D Systems AF3688), Neurofilament H (1∶1000, Millipore AB5539), and Sox2 (1∶500, Millipore AB5603). Whole cochleae were blocked in PBS with 1% Triton X-100 and 5% normal donkey serum for one hour, followed by a 20 hour incubation at 37°C in primary antibodies diluted in blocking solution at the following concentrations: Choline acetyltransferase (1∶200, Millipore AB144P), Neurofilament H (1∶1000), and Synaptophysin (1∶200, Synaptic Systems 101011). Alexa-conjugated secondary antibodies were used for signal detection. Tissue was imaged on an Olympus Fluoview FV1000 confocal microscope or a Nikon E800 compound microscope. For wholemount cochleae, the middle turn of the cochlea was imaged. The overall pattern of innervation in each image was scored as either “normal”, “intermediate”, or “abnormal” by three observers blind to genotype.

### Assessment of inner ear function

Auditory brainstem recordings (ABRs) and distortion product otoacoustic emissions (DPOAE) recordings were performed on the right ears (unless otherwise indicated) of mice at 6 weeks of age in a soundproof chamber maintained at 32°C. Prior to recordings, mutant mice were observed for circling behavior. Mice were anesthetized with ketamine (100 mg/kg) and xylazine (10 mg/kg) prior to recordings, which were performed as previously described [46]. Littermate control animals were included in each round of recordings. Due to the high lethality rate of *Lrig1^−/−^;Lrig2^−/−^* double mutants, animals used for recordings were generated by *Lrig1^+/−^;RST656^+/−^* intercrosses as well as crosses using *Lrig1^+/−^;RST656^−/−^* or *Lrig1^−/−^;RST656^+/−^* animals. Additionally, recordings were made from both ears of the *Lrig1^−/−^;Lrig2^−/−^* double mutants. Average ABR waveforms were plotted using MATLAB (MathWorks) and a script written by Ann E. Hickox in the laboratory of Dr. Charles Liberman (EPL Laboratories, Massachusetts Eye and Ear Infirmary, Boston, MA).

### Paintfilling

E14.5 mouse heads were fixed overnight at 4°C with Bodian's Fix, dehydrated in 100% ethanol, and then cleared overnight in methyl salicylate. Heads were hemisected, and White All Purpose Correction Fluid (Sanford Corporation) diluted in methyl salicylate was injected into the cochlea with a pulled glass pipette and Hamilton syringe. Filled ears were imaged in methyl salicylate using an Olympus MVX10 microscope to capture image stacks at approximately 30 µm intervals through the ear. Image stacks were processed using Image J software [47] and the Stack Focuser plugin (author Michael Umorin) to produce a single image representation (Figure 3).

## Supporting Information

Figure S1 Gene trap insertions in *Lrig1* and *Lrig2* loci. (A, B) Mutant mouse strains containing stable gene trap insertions in either *Lrig1* or *Lrig2* were utilized. In mouse line GST4169C6, insertion of the vector between exons 3 and 4 corresponds to a protein fusion to the 3rd LRR in the ectodomain (A). Similarly, insertion of a gene trap vector into exon 11 of *Lrig2* in mouse line RST656 results in a fusion to the 15th LRR of the ectodomain (B). (C) Lrig2 expression is severely reduced in *Lrig2* gene trap homozygotes, as detected in Western blots of control and mutant embryonic lysates using Lrig2 polyclonal antibody. Overexposure of the blot suggests there is only residual Lrig2 expression, which is typical of gene trap insertions. Actin was used as a loading control.
(TIF)

Figure S2 Lrig2-βgeo activity reveals broad expression of Lrig2 throughout development. Tissue from *Lrig2* heterozygotes was stained with X-gal to reveal Lrig2-βgeo activity. (A) E10.5 embryo viewed laterally. Lrig2-βgeo is active broadly, including in the early otic vesicle and cochlear vestibular ganglion. (B) Transverse section through E16.5 inner ear. Medial is to the right. Lrig2-βgeo activity was detected throughout the inner ear, with expression in all auditory and vestibular epithelia and in the spiral ganglion neurons. (C) P7 wholemount ear, viewed medially. Lrig2-βgeo activity was sustained throughout the vestibular (dashed bracket) and auditory (bracket) portions of the ear, with enhanced expression in vestibular and spiral ganglion neurons. (D, E) Transverse sections through the cochlea at E12.5 (D) and E16.5 (E). Lrig2-βgeo was active throughout the cochlear epithelium at both stages (asterisk), with enhanced expression in the spiral ganglion and low levels in the surrounding mesenchyme. (F) Wholemount P7 cochlea dissected from the inner ear. Lrig2-βgeo activity was detected in the spiral lamina and throughout the organ of Corti, with higher levels in the spiral ganglion. (G, H) Transverse sections through the vestibular organs at E16.5. As in the cochlea, Lrig2-βgeo was broadly active, indicating expression throughout the sensory and non-sensory epithelia of the utricle (G), crista (G), and saccule (H), as well as in the vestibular ganglion neurons (H). (I) Wholemount stained vestibular organs dissected from a P7 inner ear. Lrig2-βgeo activity persisted, with high levels in the sensory epithelia in the utricle and in the cristae. ac = anterior crista, c = crista, cvg = cochlear vestibular ganglion, g = gut, lc = lateral crista, nt = neural tube, oC = organ of Corti, ov = otic vesicle, pc = posterior crista, s = saccule, sg = spiral ganglion, sl = spiral lamina, sm = somite, ssc = semicircular canal, tg = trigeminal ganglion, u = utricle, vg = vestibular ganglion. Scale bar = 50 µm.
(TIF)

Figure S3 Validation of Lrig1 polyclonal antibody using control and *Lrig1* mutant tissue. Transverse sections through the inner ear of E18.5 control (A, C, E) and *Lrig1^−/−^;Lrig2^+/−^* animals (B, D, F) immunostained for Lrig1 and Myo7a and counterstained with DAPI. The Lrig1 channel is shown on its own in A′–F′ for easier visualization. The gross structure of the vestibular (A–D) and auditory (E, F) sections of the inner ear was unchanged in *Lrig1* mutant mice when compared to controls. Lrig1 protein was detected in the non-sensory epithelium of the utricle and saccule (A), in projections to the utricle and lateral crista (C), and in the medial wall of the cochlea (E). This staining was lost in mutants (B, D, F), confirming that this antibody specifically detects Lrig1 and not other family members. In addition, this result indicates that Lrig1 protein is severely reduced in the gene trap mutant. c = crista, oC = organ of Corti, s = saccule, sg = spiral ganglion, st = scala tympani, sv = scala vestibuli, u = utricle. Scale bar = 40 µm.
(TIF)

Figure S4 Analysis of cochlear morphology and innervation. (A–D) Transverse sections through the inner ears of E19 control (A, C) and double mutant (B, D) animals were immunostained to visualize neurons (NF) and hair cells (Myo7a) and counterstained with DAPI (A, B). The cochlea was histologically normal in double mutants at a gross level (compare A and B). There were no obvious changes in the structure of the duct (the apical turn is at the top), spiral ganglion neurons were present in each turn of the cochlea (asterisks), and a well-defined eighth nerve was present (VIII). Further, closer examination (region in box, A), showed innervation of Myo7a-positive hair cells in the organ of Corti by NF-positive projections from the spiral ganglion (C, D). NF staining of whole cochleae from *Lrig1^−/−^* (F), *Lrig2^−/−^* (G), and *Lrig1^+/−^;Lrig2^−/−^* (H) adult animals revealed no obvious changes in the pattern of innervation compared to control animals (E). Efferent innervation was also unaffected, as shown by staining for choline acetyltransferase (ChAT) (F′, H′). Scale bar = 50 µm.
(TIF)

Table S1 Threshold values for ABR and DPOAE recordings. Values indicate average thresholds (in decibels) ± standard error of the mean as determined by recording DPOAEs (top) or ABRs (bottom) in response to stimuli across a range of frequencies (in kilohertz (kHz)). *Lrig1^−/−^* mutant animals showed elevated thresholds relative to control animals (*Lrig1* ^+*/−*^;*Lrig2^+/−^*), but *Lrig2^−/−^* responses were unaffected. The additional loss of *Lrig2* further increased the threshold response of *Lrig1^−/−^* mutants (compare *Lrig1^−/−^;Lrig2^+/−^* vs. *Lrig1^−/−^;Lrig2^−/−^* values).
(DOCX)

Table S2 Statistical significance of differences in DPOAE and ABR values across frequencies. Columns indicate *P* values obtained by Student's t-test comparison of DPOAE thresholds, ABR thresholds, ABR latencies, and the maximum amplitude of the first peak in the ABR response, with significance shown across a range of frequencies in animals of each genotype vs. wild-types. A value of 0.05 or lower was considered significant. *Lrig1^−/−^;Lrig2^−/−^* double mutant animal responses were significantly different from wild-types for all parameters at all frequencies tested. NS = not significant.
(DOCX)

Table S3 ABR amplitude values at 80 dB sound intensity stimulation. Values represent the average amplitude (in microvolts) ± standard error of the mean of the first peak in the ABR response to 80 dB stimuli across a range of frequencies. *Lrig1^−/−^;Lrig2^−/−^* double mutant animal responses show severely decreased amplitudes across all frequencies tested.
(DOCX)

Table S4 Statistical significance of differences in ABR amplitude at 16 kHz. Columns indicate *P* values obtained by comparing ABR amplitudes in animals for each genotype vs. wild-type. Responses to a range of stimulus intensities (30 to 80 decibels (dB)) were compared. A value of 0.05 or lower was considered significant. *Lrig1^−/−^;Lrig2^+/−^* and *Lrig1^−/−^;Lrig2^−/−^* mutant animal responses were significantly different across all intensities tested. NS = not significant.
(DOCX)

Video S1 Circling behavior in *Lrig1^−/−^;Lrig2^−/−^* double mutant. A movie illustrating circling behavior in a 6 week old *Lrig1^−/−^;Lrig2^−/−^* female.
(MOV)
