# Supplementary figures and images for: In Vivo Analysis of Lrig Genes Reveals Redundant and Independent Functions in the Inner Ear
Source: PLoS Genet. 2013 Sep 26;9(9):e1003824. doi: 10.1371/journal.pgen.1003824 (PMC3784559; doi:10.1371/journal.pgen.1003824)

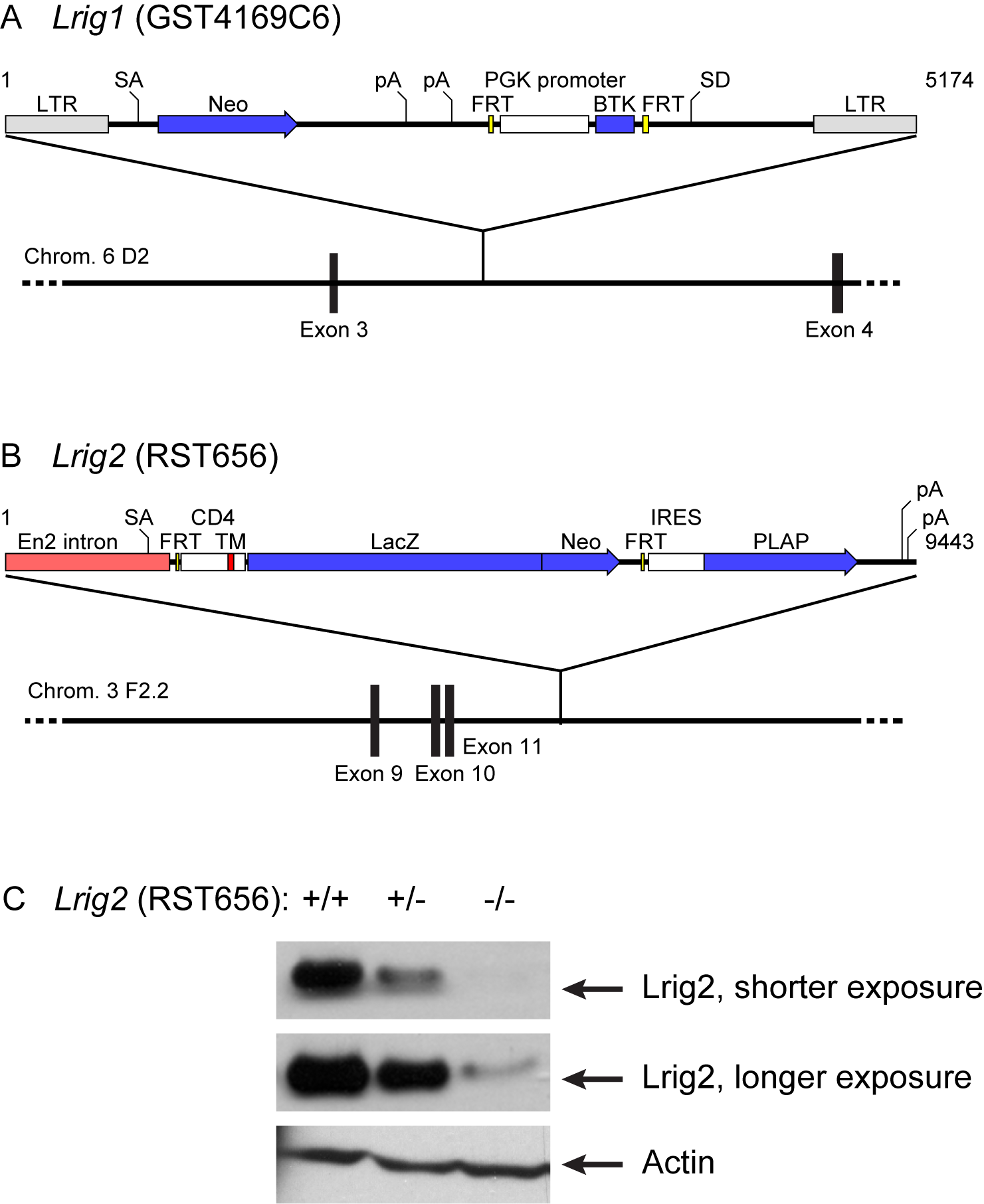

Supplement: Figure S1 — Gene trap insertions in Lrig1 and Lrig2 loci. (A, B) Mutant mouse strains containing stable gene trap insertions in either Lrig1 or Lrig2 were utilized. In mouse line GST4169C6, insertion of the vector between exons 3 and 4 corresponds to a protein fusion to the 3rd LRR in the ectodomain (A). Similarly, insertion of a gene trap vector into exon 11 of Lrig2 in mouse line RST656 results in a fusion to the 15th LRR of the ectodomain (B). (C) Lrig2 expression is severely reduced in Lrig2 gene trap homozygotes, as detected in Western blots of control and mutant embryonic lysates using Lrig2 polyclonal antibody. Overexposure of the blot suggests there is only residual Lrig2 expression, which is typical of gene trap insertions. Actin was used as a loading control. (TIF) [file pgen.1003824.s001.tif]

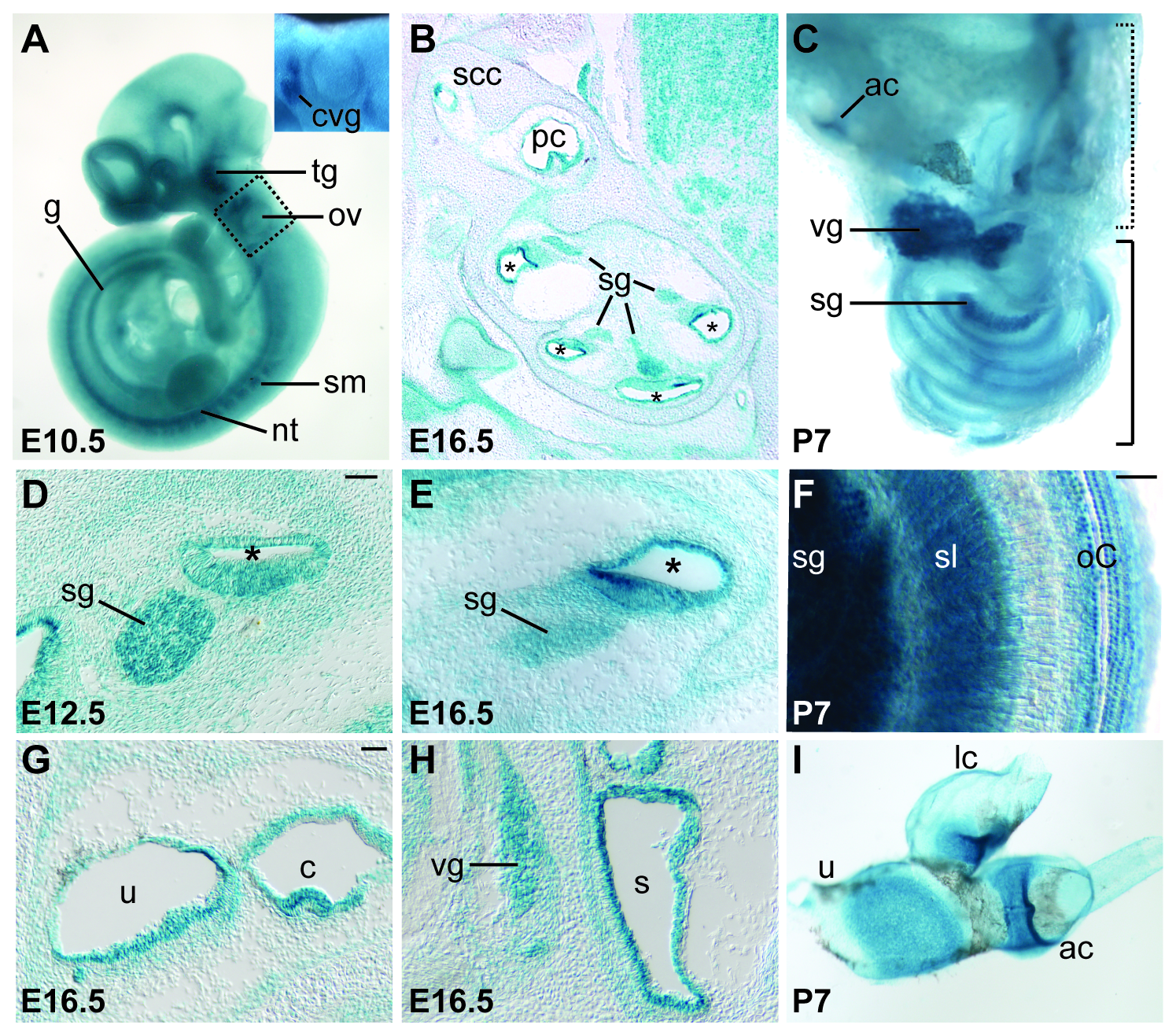

Supplement: Figure S2 — Lrig2-βgeo activity reveals broad expression of Lrig2 throughout development. Tissue from Lrig2 heterozygotes was stained with X-gal to reveal Lrig2-βgeo activity. (A) E10.5 embryo viewed laterally. Lrig2-βgeo is active broadly, including in the early otic vesicle and cochlear vestibular ganglion. (B) Transverse section through E16.5 inner ear. Medial is to the right. Lrig2-βgeo activity was detected throughout the inner ear, with expression in all auditory and vestibular epithelia and in the spiral ganglion neurons. (C) P7 wholemount ear, viewed medially. Lrig2-βgeo activity was sustained throughout the vestibular (dashed bracket) and auditory (bracket) portions of the ear, with enhanced expression in vestibular and spiral ganglion neurons. (D, E) Transverse sections through the cochlea at E12.5 (D) and E16.5 (E). Lrig2-βgeo was active throughout the cochlear epithelium at both stages (asterisk), with enhanced expression in the spiral ganglion and low levels in the surrounding mesenchyme. (F) Wholemount P7 cochlea dissected from the inner ear. Lrig2-βgeo activity was detected in the spiral lamina and throughout the organ of Corti, with higher levels in the spiral ganglion. (G, H) Transverse sections through the vestibular organs at E16.5. As in the cochlea, Lrig2-βgeo was broadly active, indicating expression throughout the sensory and non-sensory epithelia of the utricle (G), crista (G), and saccule (H), as well as in the vestibular ganglion neurons (H). (I) Wholemount stained vestibular organs dissected from a P7 inner ear. Lrig2-βgeo activity persisted, with high levels in the sensory epithelia in the utricle and in the cristae. ac = anterior crista, c = crista, cvg = cochlear vestibular ganglion, g = gut, lc = lateral crista, nt = neural tube, oC = organ of Corti, ov = otic vesicle, pc = posterior crista, s = saccule, sg = spiral ganglion, sl = spiral lamina, sm = somite, ssc = semicircular canal, tg = trigeminal ganglion, u = utricle, vg = vestibular ganglion. [file pgen.1003824.s002.tif]

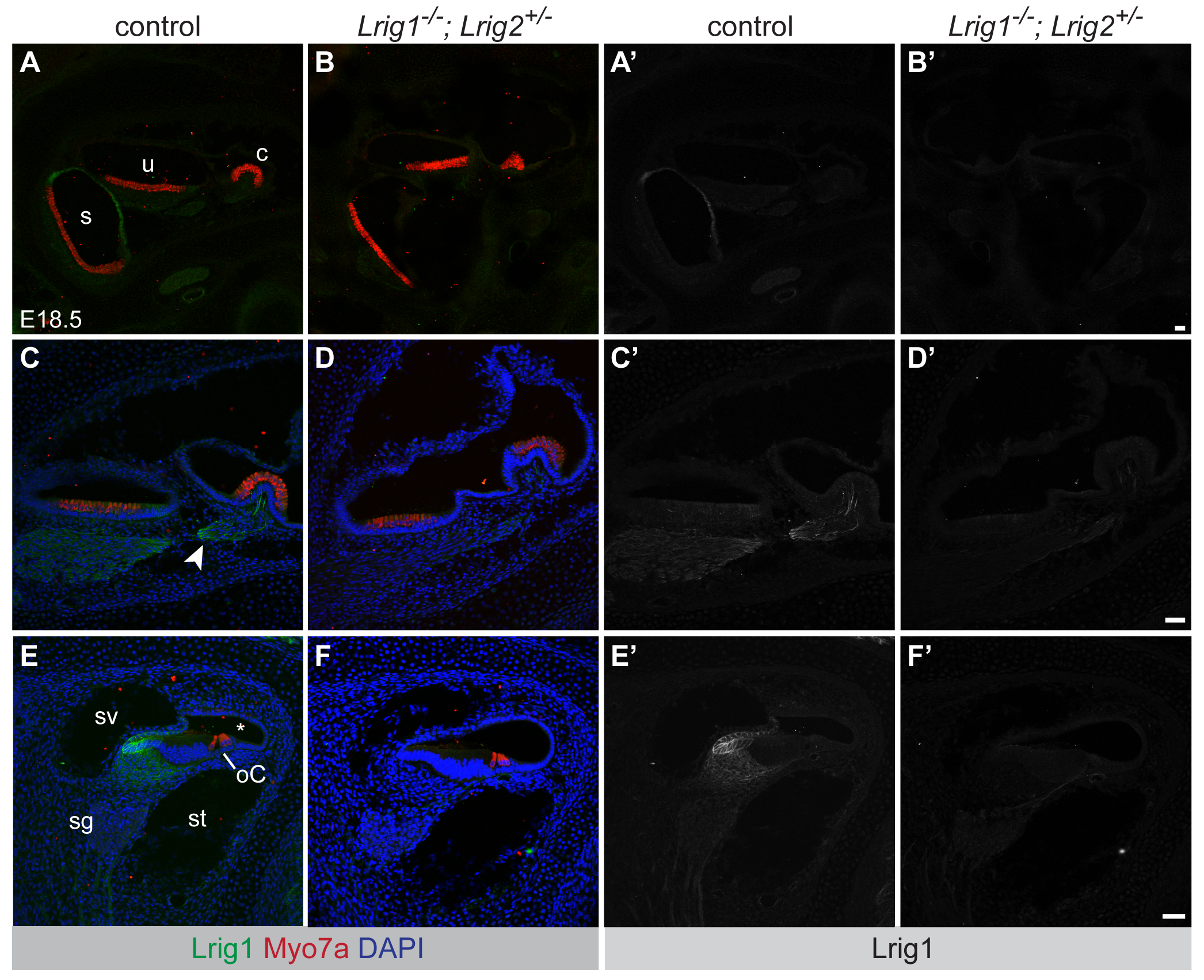

Supplement: Figure S3 — Validation of Lrig1 polyclonal antibody using control and Lrig1 mutant tissue. Transverse sections through the inner ear of E18.5 control (A, C, E) and Lrig1−/−;Lrig2+/− animals (B, D, F) immunostained for Lrig1 and Myo7a and counterstained with DAPI. The Lrig1 channel is shown on its own in A′–F′ for easier visualization. The gross structure of the vestibular (A–D) and auditory (E, F) sections of the inner ear was unchanged in Lrig1 mutant mice when compared to controls. Lrig1 protein was detected in the non-sensory epithelium of the utricle and saccule (A), in projections to the utricle and lateral crista (C), and in the medial wall of the cochlea (E). This staining was lost in mutants (B, D, F), confirming that this antibody specifically detects Lrig1 and not other family members. In addition, this result indicates that Lrig1 protein is severely reduced in the gene trap mutant. c = crista, oC = organ of Corti, s = saccule, sg = spiral ganglion, st = scala tympani, sv = scala vestibuli, u = utricle. Scale bar = 40 µm. (TIF) [file pgen.1003824.s003.tif]

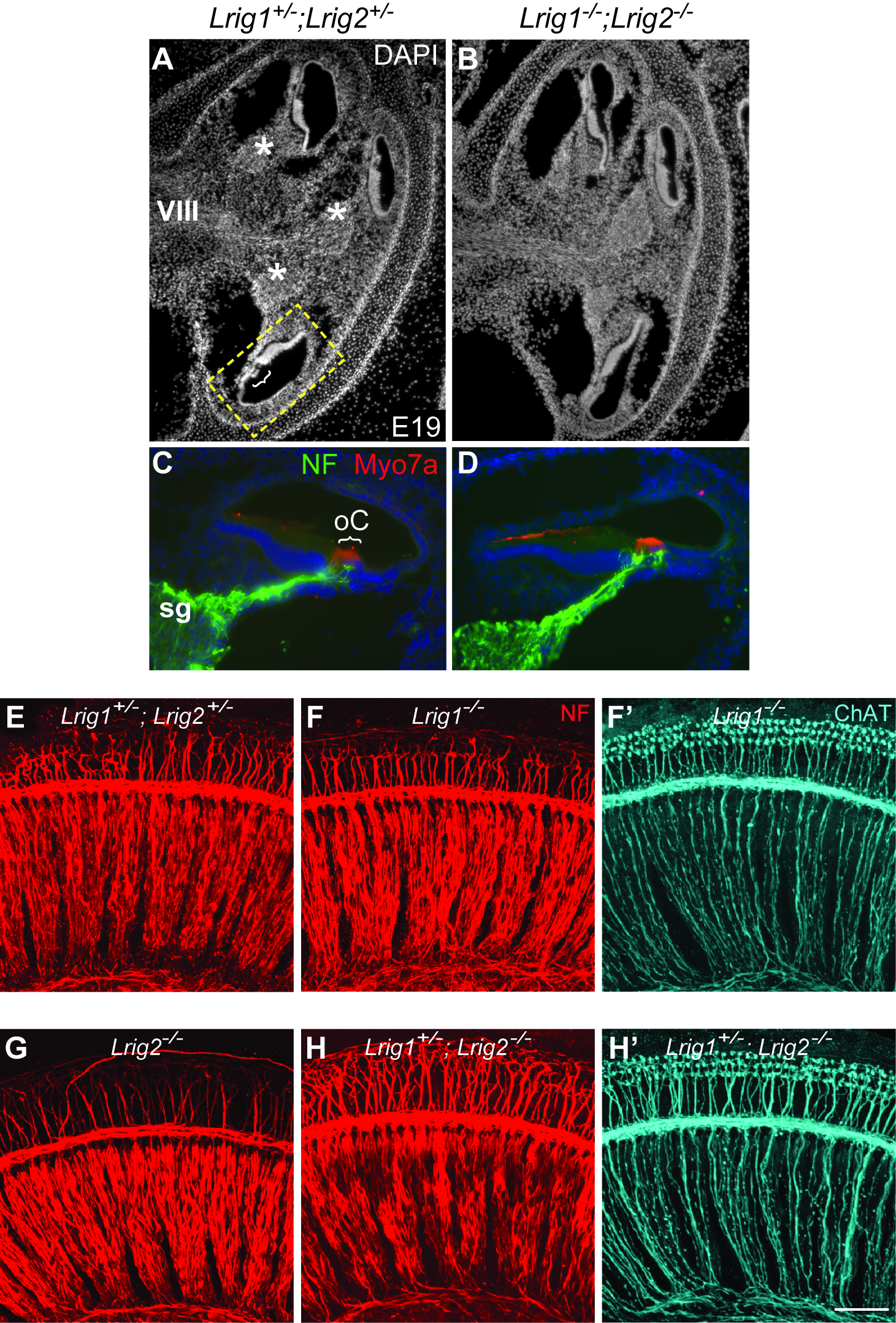

Supplement: Figure S4 — Analysis of cochlear morphology and innervation. (A–D) Transverse sections through the inner ears of E19 control (A, C) and double mutant (B, D) animals were immunostained to visualize neurons (NF) and hair cells (Myo7a) and counterstained with DAPI (A, B). The cochlea was histologically normal in double mutants at a gross level (compare A and B). There were no obvious changes in the structure of the duct (the apical turn is at the top), spiral ganglion neurons were present in each turn of the cochlea (asterisks), and a well-defined eighth nerve was present (VIII). Further, closer examination (region in box, A), showed innervation of Myo7a-positive hair cells in the organ of Corti by NF-positive projections from the spiral ganglion (C, D). NF staining of whole cochleae from Lrig1−/− (F), Lrig2−/− (G), and Lrig1+/−;Lrig2−/− (H) adult animals revealed no obvious changes in the pattern of innervation compared to control animals (E). Efferent innervation was also unaffected, as shown by staining for choline acetyltransferase (ChAT) (F′, H′). Scale bar = 50 µm. (TIF) [file pgen.1003824.s004.tif]
