# Supplementary material for: In Vivo Analysis of Lrig Genes Reveals Redundant and Independent Functions in the Inner Ear
Source: PLoS Genet. 2013 Sep 26;9(9):e1003824. doi: 10.1371/journal.pgen.1003824 (PMC3784559; doi:10.1371/journal.pgen.1003824)
Supplement: Table S1 — Threshold values for ABR and DPOAE recordings. Values indicate average thresholds (in decibels) ± standard error of the mean as determined by recording DPOAEs (top) or ABRs (bottom) in response to stimuli across a range of frequencies (in kilohertz (kHz)). Lrig1−/− mutant animals showed elevated thresholds relative to control animals (Lrig1 +/−;Lrig2+/−), but Lrig2−/− responses were unaffected. The additional loss of Lrig2 further increased the threshold response of Lrig1−/− mutants (compare Lrig1−/−;Lrig2+/− vs. Lrig1−/−;Lrig2−/− values). (DOCX) [file pgen.1003824.s005.docx]

**Table S1. Threshold values for ABR and DPOAE recordings.**

| ***Lrig1; Lrig2* genotype** | **5.6 kHz** | **8 kHz** | **11.3 kHz** | **16 kHz** | **22.6 kHz** | **32 kHz** |
| --- | --- | --- | --- | --- | --- | --- |
| **DPOAE Threshold** | | | | | | |
| ***+/-; +/-*** | 73.24 ± 2.31 | 64.65 ± 4.27 | 29.78 ± 1.93 | 16.51 ± 1.95 | 41.01 ± 3.65 | 56.65 ± 4.40 |
| ***+/+; -/-*** | 70.14 ± 2.48 | 68.96 ± 4.47 | 31.76 ± 4.25 | 11.76 ± 2.89 | 47.81 ± 4.13 | 60.32 ± 4.24 |
| ***+/-; -/-*** | 68.15 ± 4.80 | 73.16 ± 2.98 | 32.75 ± 3.12 | 13.93 ± 3.28 | 41.31 ± 6.44 | 58.45 ± 4.98 |
| ***-/-; +/+*** | 78.02 ± 3.19 | 76.86 ± 6.48 | 55.20 ± 11.48 | 44.69 ± 13.48 | 61.32 ± 7.21 | 71.86 ± 4.50 |
| ***-/-; +/-*** | 78.66 ± 1.87 | 82.67 ± 1.54 | 62.35 ± 6.61 | 57.37 ± 8.72 | 63.85 ± 5.83 | 64.17 ± 4.83 |
| ***-/-; -/-*** | 78.23 ± 1.71 | 82.87 ± 2.13 | 66.50 ± 7.00 | 55.79 ± 7.52 | 63.68 ± 6.12 | 68.59 ± 2.83 |
| **ABR Threshold** | | | | | | |
| ***+/-; +/-*** | 31.25 ± 2.62 | 22.92 ± 2.50 | 17.92 ± 1.68 | 17.14 ± 2.60 | 22.92 ± 4.37 | 24.55 ± 3.27 |
| ***+/+; -/-*** | 39.38 ± 4.57 | 28.13 ± 2.98 | 20.00 ± 3.54 | 16.25 ± 2.45 | 31.25 ± 5.49 | 30.63 ± 5.30 |
| ***+/-; -/-*** | 41.25 ± 3.24 | 28.13 3.40 | 20.63 ± 1.75 | 23.13 ± 5.72 | 22.50 ± 5.98 | 28.13 ± 6.40 |
| ***-/-; +/+*** | 51.00 ± 9.27 | 55.00 ± 10.37 | 28.00 ± 7.52 | 34.00 ± 8.28 | 47.00 ± 6.44 | 44.00 ± 5.79 |
| ***-/-; +/-*** | 58.75 ± 7.89 | 60.00 ± 8.24 | 45.63 ± 5.78 | 42.50 ± 6.12 | 46.25 ± 4.51 | 43.13 ± 5.74 |
| ***-/-; -/-*** | 71.50 ± 5.58 | 64.50 ± 5.02 | 48.50 ± 6.46 | 53.50 ± 6.01 | 50.50 ± 3.91 | 49.00 ± 4.52 |
