# Supplementary material for: In Vivo Analysis of Lrig Genes Reveals Redundant and Independent Functions in the Inner Ear
Source: PLoS Genet. 2013 Sep 26;9(9):e1003824. doi: 10.1371/journal.pgen.1003824 (PMC3784559; doi:10.1371/journal.pgen.1003824)
Supplement: Table S2 — Statistical significance of differences in DPOAE and ABR values across frequencies. Columns indicate P values obtained by Student's t-test comparison of DPOAE thresholds, ABR thresholds, ABR latencies, and the maximum amplitude of the first peak in the ABR response, with significance shown across a range of frequencies in animals of each genotype vs. wild-types. A value of 0.05 or lower was considered significant. Lrig1−/−;Lrig2−/− double mutant animal responses were significantly different from wild-types for all parameters at all frequencies tested. NS = not significant. (DOCX) [file pgen.1003824.s006.docx]

**Table S2. Statistical significance of differences in DPOAE and ABR values across frequencies.**

| ***Lrig1; Lrig2* genotype** | **5.6 kHz** | **8 kHz** | **11.3 kHz** | **16 kHz** | **22.6 kHz** | **32 kHz** |
| --- | --- | --- | --- | --- | --- | --- |
| **DPOAE Threshold** | | | | | | |
| ***+/-; +/-*** | NS | NS | NS | NS | NS | NS |
| ***+/+; -/-*** | NS | NS | NS | NS | NS | NS |
| ***+/-; -/-*** | NS | NS | NS | NS | NS | NS |
| ***-/-; +/+*** | NS | NS | 0.010 | 0.008 | 0.035 | NS |
| ***-/-; +/-*** | 0.035 | 0.003 | 0.0001 | 0.0001 | 0.009 | NS |
| ***-/-; -/-*** | 0.042 | 0.002 | 0.00003 | 0.0001 | 0.013 | 0.007 |
| **ABR Threshold** | | | | | | |
| ***+/-; +/-*** | NS | NS | NS | NS | NS | NS |
| ***+/+; -/-*** | NS | NS | NS | NS | NS | NS |
| ***+/-; -/-*** | NS | NS | 0.004 | NS | NS | NS |
| ***-/-; +/+*** | NS | 0.001 | 0.019 | 0.013 | NS | NS |
| ***-/-; +/-*** | 0.008 | 0.0002 | 0.00002 | 0.0003 | 0.029 | NS |
| ***-/-; -/-*** | 0.00001 | 0.000001 | 0.00001 | 0.00001 | 0.004 | 0.005 |
| **ABR Latency** | | | | | | |
| ***+/-; +/-*** | NS | NS | NS | NS | NS | NS |
| ***+/+; -/-*** | 0.004 | 0.0003 | 0.026 | NS | NS | NS |
| ***+/-; -/-*** | 0.0001 | 0.001 | 0.002 | 0.009 | NS | NS |
| ***-/-; +/+*** | NS | 0.027 | 0.044 | 0.031 | 0.005 | NS |
| ***-/-; +/-*** | 0.017 | 0.011 | 0.026 | 0.006 | 0.024 | NS |
| ***-/-; -/-*** | 0.014 | 0.010 | 0.000004 | 0.00002 | 0.0001 | 0.001 |
| **Max. ABR Amplitude** | | | | | | |
| ***+/-; +/-*** | NS | NS | NS | NS | NS | NS |
| ***+/+; -/-*** | 0.029 | 0.0003 | 0.002 | 0.009 | NS | NS |
| ***+/-; -/-*** | 0.001 | 0.002 | 0.004 | 0.005 | NS | 0.019 |
| ***-/-; +/+*** | NS | 0.028 | NS | NS | 0.030 | 0.019 |
| ***-/-; +/-*** | 0.004 | 0.00004 | 0.002 | 0.001 | NS | NS |
| ***-/-; -/-*** | 0.00001 | <0.000001 | 0.00001 | 0.000005 | 0.0005 | 0.007 |
