# Supplementary material for: In Vivo Analysis of Lrig Genes Reveals Redundant and Independent Functions in the Inner Ear
Source: PLoS Genet. 2013 Sep 26;9(9):e1003824. doi: 10.1371/journal.pgen.1003824 (PMC3784559; doi:10.1371/journal.pgen.1003824)
Supplement: Table S3 — ABR amplitude values at 80 dB sound intensity stimulation. Values represent the average amplitude (in microvolts) ± standard error of the mean of the first peak in the ABR response to 80 dB stimuli across a range of frequencies. Lrig1−/−;Lrig2−/− double mutant animal responses show severely decreased amplitudes across all frequencies tested. (DOCX) [file pgen.1003824.s007.docx]

**Table S3. ABR amplitude values at 80 dB sound intensity stimulation.**

| ***Lrig1; Lrig2* genotype** | **5.6 kHz** | **8 kHz** | **11.3 kHz** | **16 kHz** | **22.6 kHz** | **32 kHz** |
| --- | --- | --- | --- | --- | --- | --- |
| **ABR Amplitude** | | | | | | |
| ***+/-; +/-*** | 1.40 ± 0.19 | 1.95 ± 0.25 | 2.72 ± 0.24 | 2.83 ± 0.28 | 2.60 ± 0.32 | 1.60 ± 0.22 |
| ***+/+; -/-*** | 0.71 ± 0.12 | 0.94 ± 0.15 | 1.70 ± 0.20 | 2.22 ± 0.23 | 1.41 ± 0.15 | 1.15 ± 0.18 |
| ***+/-; -/-*** | 0.53 ± 0.08 | 1.08 ± 0.16 | 1.80 ± 0.19 | 2.04 ± 0.34 | 1.67 ± 0.34 | 0.85 ± 0.16 |
| ***-/-; +/+*** | 0.78 ± 0.31 | 0.99 ± 0.40 | 2.17 ± 0.42 | 2.34 ± 0.42 | 1.11 ± 0.20 | 1.06 ± 0.11 |
| ***-/-; +/-*** | 0.48 ± 0.15 | 0.56 ± 0.20 | 1.47 ± 0.31 | 1.81 ± 0.26 | 1.53 ± 0.19 | 1.22 ± 0.18 |
| ***-/-; -/-*** | 0.22 ± 0.11 | 0.29 ± 0.12 | 0.82 ± 0.26 | 0.88 ± 0.29 | 0.70 ± 0.19 | 0.66 ± 0.21 |
