# Supplementary material for: In Vivo Analysis of Lrig Genes Reveals Redundant and Independent Functions in the Inner Ear
Source: PLoS Genet. 2013 Sep 26;9(9):e1003824. doi: 10.1371/journal.pgen.1003824 (PMC3784559; doi:10.1371/journal.pgen.1003824)
Supplement: Table S4 — Statistical significance of differences in ABR amplitude at 16 kHz. Columns indicate P values obtained by comparing ABR amplitudes in animals for each genotype vs. wild-type. Responses to a range of stimulus intensities (30 to 80 decibels (dB)) were compared. A value of 0.05 or lower was considered significant. Lrig1−/−;Lrig2+/− and Lrig1−/−;Lrig2−/− mutant animal responses were significantly different across all intensities tested. NS = not significant. (DOCX) [file pgen.1003824.s008.docx]

**Table S4. Statistical significance of differences in ABR amplitude at 16 kHz.**

| ***Lrig1; Lrig2* genotype** | **30 dB** | **40 dB** | **50 dB** | **60 dB** | **70 dB** | **80 dB** |
| --- | --- | --- | --- | --- | --- | --- |
| ***+/-; +/-*** | NS | NS | NS | NS | NS | NS |
| ***+/+; -/-*** | NS | 0.005 | 0.007 | 0.005 | 0.0009 | 0.009 |
| ***+/-; -/-*** | NS | 0.007 | 0.014 | 0.037 | 0.006 | 0.006 |
| ***-/-; +/+*** | NS | 0.015 | 0.015 | 0.016 | 0.022 | NS |
| ***-/-; +/-*** | 0.002 | 0.00005 | 0.0006 | 0.0004 | 0.00009 | 0.0008 |
| ***-/-; -/-*** | 0.0001 | 0.000004 | 0.000003 | 0.000001 | <0.000001 | 0.000005 |
